# E‐52862—A selective sigma‐1 receptor antagonist, in peripheral neuropathic pain: Two randomized, double‐blind, phase 2 studies in patients with chronic postsurgical pain and painful diabetic neuropathy

**DOI:** 10.1002/ejp.4755

**Published:** 2024-12-04

**Authors:** Rafael Gálvez, Victor Mayoral, Jesús Cebrecos, Francisco J. Medel, Adelaida Morte, Mariano Sust, Anna Vaqué, Antonio Montes‐Pérez, Fernando Neira‐Reina, Luz Cánovas, César Margarit, Didier Bouhassira

**Affiliations:** ^1^ Pain Clinic Virgen de Las Nieves University Hospital Granada Spain; ^2^ Bellvitge University Hospital L'Hospitalet de Llobregat Barcelona Spain; ^3^ ESTEVE Pharmaceuticals S.A. Barcelona Spain; ^4^ Pain Unit Vall d'Hebron Research Institute Barcelona Spain; ^5^ Pain Unit Hospital del Mar Research Institute Barcelona Spain; ^6^ Puerto Real University Hospital Cádiz Spain; ^7^ Pain Clinic Ourense University Hospital Complex (CHUO) Ourense Spain; ^8^ Pain Unit Alicante University General Hospital Alicante Spain; ^9^ Inserm U987, UVSQ Paris‐Saclay University, Ambroise‐Paré Hospital Boulogne‐Billancourt France

## Abstract

**Background:**

We report the efficacy and safety of E‐52862—a selective, sigma‐1 receptor antagonist—from phase 2, randomized, proof‐of‐concept studies in patients with moderate‐to‐severe, neuropathic, chronic postsurgical pain (CPSP) and painful diabetic neuropathy (PDN).

**Methods:**

Adult patients (CPSP [*N* = 116]; PDN [*N* = 163]) were randomized at a 1:1 ratio to 4 weeks of treatment with E‐52862 (CPSP [*n* = 55]; PDN [*n* = 85]) or placebo (CPSP [*n* = 61]; PDN [*n* = 78]) orally once daily. Pain intensity scores were measured using a numerical pain rating scale from 0 (no pain) to 10 (worst pain imaginable). The primary analysis population comprised patients who received study drug with ≥1 baseline and on‐treatment observation (full analysis set).

**Results:**

In CPSP, mean baseline average pain was 6.2 for E‐52862 vs. 6.5 for placebo. Week 4 mean change from baseline (CFB) for average pain was −1.6 for E‐52862 vs. –0.9 for placebo (least squares mean difference [LSMD]: −0.9; *p* = 0.029). In PDN, mean baseline average pain was 5.3 for E‐52862 vs. 5.4 for placebo. Week 4 mean CFB for average pain was −2.2 for E‐52862 vs. –2.1 for placebo (LSMD: –0.1; *p* = 0.766). Treatment‐emergent adverse events (TEAEs) were reported in 90.9% of E‐52862‐treated patients vs. 76.7% of placebo‐treated patients in CPSP and 34.1% vs. 26.9% in PDN. Serious TEAEs occurred in CPSP only: E‐52862: 5.5%; placebo: 6.7%.

**Conclusions:**

E‐52862 demonstrated superior relief of CPSP vs. placebo after 4 weeks. Reductions in pain intensity were seen in PDN with E‐52862; high placebo response rates may have prevented differentiation between treatments. E‐52862 had acceptable tolerability in both populations.

**Significance Statement:**

These proof‐of‐concept studies validate the mode of action of E‐52862, a selective sigma‐1 receptor antagonist. In CPSP, E‐52862 resulted in clinically meaningful pain relief. In PDN, reductions in pain intensity were seen with E‐52862; high placebo response rates may have prevented differentiation between E‐52862 and placebo. These findings are clinically relevant given that neuropathic pain is highly incapacitating, lacking effective treatments and representing a significant unmet medical need, and support further development of sigma‐1 receptor antagonists for peripheral neuropathic pain.

## 1 INTRODUCTION

Peripheral neuropathic pain represents a major unmet clinical need. It affects approximately 7%–10% of the general population (Bouhassira et al., 2008; van Hecke et al., 2014), but is more prevalent among people who undergo surgery (Rosenberger & Pogatzki‐Zahn, 2022) and those with diabetes (Bouhassira et al., 2013; Selvarajah et al., 2019; Tesfaye et al., 2011). Over half of patients do not respond to current treatments (Bouhassira & Attal, 2023; Finnerup et al., 2015; Moisset et al., 2021); management is therefore challenging and often suboptimal, impairing quality of life (Colloca et al., 2017).

Chronic postsurgical pain (CPSP), a common surgical complication, is often underdiagnosed and poorly treated (Giaccari et al., 2021; Montes et al., 2015; Rosenberger & Pogatzki‐Zahn, 2022). Risk of CPSP varies based on the type of tissue injury, and the site, extent, duration and type of surgery. Procedures associated with a high risk include knee and hip arthroplasty, thoracotomy, amputation and coronary artery bypass grafting surgery (Charlton et al., 2024; Richebé et al., 2018; Rosenberger & Pogatzki‐Zahn, 2022; Schug et al., 2019). CPSP shows neuropathic pain characteristics in up to 80% of cases with certain surgeries (Rosenberger & Pogatzki‐Zahn, 2022; Schug et al., 2019).

Prevalence of painful diabetic neuropathy (PDN) among patients with diabetes ranges from 8% to 35%, and PDN impacts quality of life (Abbott et al., 2011; Gylfadottir et al., 2020; Risson et al., 2017; Staehelin, 2023; Syed et al., 2023; Ziegler et al., 2022). Treatment of PDN is challenging, and it remains underdiagnosed and undertreated (Jensen et al., 2021; Sloan et al., 2021; Syed et al., 2023).

The calcium ion‐sensing chaperone protein sigma‐1 receptor (S1R) regulates several processes, including intracellular signalling cascades related to noxious stimuli and nociception (Ruiz‐Cantero et al., 2021). S1R is expressed in areas important for pain control in the central and peripheral nervous systems and physically interacts with several membrane proteins including *N*‐methyl*‐D*‐aspartate receptors; in S1R‐knockout mice, neuropathic pain‐like behaviours were attenuated (Rabiner et al., 2022). S1R emerged as an analgesic target over the last decade, with a potential role in modulating antinociception (Abadias et al., 2013). E‐52862 is a novel, selective, S1R antagonist (S1RA) that reduces nerve injury‐evoked activity of S1R‐modulated receptors involved in central (spinal) sensitization and pain hypersensitivity (Ruiz‐Cantero et al., 2021). In animal models of neuropathic pain, E‐52862 crossed the blood–brain barrier, binding to S1R in the central nervous system, demonstrating analgesic activity for neuropathic pain (Bura et al., 2013; Nieto et al., 2012; Romero et al., 2012; Zamanillo et al., 2013). Pharmacokinetic in‐human studies showed E‐52862 has rapid, dose‐dependent absorption (Abadias et al., 2013), with a monotonic relationship between plasma drug levels and brain S1R occupancy (Rabiner et al., 2022), allowing for once‐daily dosing. In phase 1 studies with healthy volunteers, multiple E‐52862 doses up to 400 mg daily had acceptable safety, tolerability, pharmacokinetic, and pharmacodynamic profiles (Abadias et al., 2013; Rabiner et al., 2022; Täubel et al., 2015), with no serious adverse events (AEs) or critical risks (Abadias et al., 2013; Rabiner et al., 2022; Täubel et al., 2015). Daily 400‐mg dosing was therefore chosen for further study.

E‐52862 was investigated for oxaliplatin‐induced peripheral neuropathy in patients with colorectal cancer receiving the folinic acid, fluorouracil, and oxaliplatin (FOLFOX) chemotherapy regimen in a randomized, multicentre, double‐blind, placebo‐controlled, phase 2 trial (Bruna et al., 2018). E‐52862 had an acceptable safety profile with evidence of a potential neuroprotective role for chronic cumulative oxaliplatin‐induced peripheral neuropathy (Bruna et al., 2018).

We report the analgesic efficacy, safety and tolerability of E‐52862 400 mg once daily (QD) from two phase 2, randomized, proof‐of‐concept studies in patients with moderate‐to‐severe CPSP and moderate‐to‐severe PDN.

## 2 METHODS

### 2.1 Study design and participants

**FIGURE 1:**
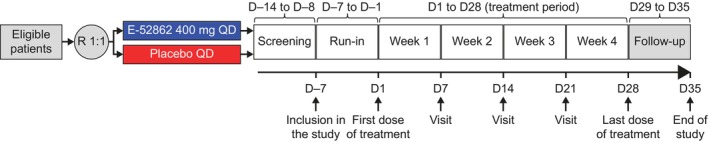
Study design for the CPSP and PDN studies. CPSP, chronic postsurgical pain; D, day; PDN, painful diabetic neuropathy; QD, once daily; R, randomization.

The CPSP Study (SIGM‐205, EudraCT Number: 2012–000402‐30) and PDN Study (SIGM‐204, EudraCT Number: 2012–000400‐14) were randomized, double‐blind, placebo‐controlled, phase 2, proof‐of‐concept studies in patients with moderate‐to‐severe CPSP and PDN, respectively. The CPSP Study was conducted at 15 centres in Spain, and the PDN Study was conducted at 26 centres across Spain and Romania. Both studies were ≤9 weeks in duration, including a screening period (up to 3 weeks), a 7‐day run‐in period, a 28‐day treatment period and a 7‐day follow‐up period (Figure 1).

Eligible patients were aged ≥18 years with the following: neuropathic CPSP caused by a surgical intervention that occurred ≥3 months before enrolment (CPSP Study), or type 1 or 2 diabetes and PDN that had been present for ≥6 months but <5 years, beginning in the feet with relatively symmetrical onset (PDN Study). Average daily pain score using a numerical pain rating scale (NPRS) had to be ≥4 at screening and during the run‐in period, with ≥5 assessments recorded. The neuropathic pain component was identified using the Douleur Neuropathique en 4 Questions (DN4; DN4 score ≥4) tool in the CPSP Study (Bouhassira et al., 2005). In the PDN Study, the Michigan Neuropathy Screening Instrument (MNSI) was used to assess peripheral neuropathy (MNSI part B score ≥3) and a NPRS to assess pain intensity (Feldman et al., 1994). Exclusion criteria included any other active medical condition that could interfere with study assessments or compromise patient safety, severe pain from other causes, a clinically relevant laboratory abnormality or use of prohibited medication. Full inclusion and exclusion criteria are listed in the Data S1.

Both studies were conducted in accordance with Good Clinical Practice, the Declaration of Helsinki and all other applicable standards, and all patients provided written informed consent. The CPSP Study protocol was approved by the Spanish Agency of Medicines and Medical Devices (AEMPS; 18 June 2012) and the Reference Ethics Committee, Hospital Clínic de Barcelona, Barcelona (8 June 2012). The PDN Study protocol was approved by the Reference Ethics Committee, Hospital Clínico San Carlos, Madrid (7 June 2012), the AEMPS (14 June 2012), the Romanian National Bioethics Committee for Medicines and Medical Devices (10 July 2014) and the National Agency for Medicines and Medical Devices of Romania (ANM; 11 July 2014).

### 2.2 Procedures

Within each trial, eligible patients were randomized at a 1:1 ratio to receive 4 weeks of treatment (until Day 28) with E‐52862 or placebo (Figure 1). The randomization methods are described in detail in the Data S1. E‐52862 400 mg and placebo QD were given orally in the morning under fasting conditions. Patients attended clinic visits once a week for assessments. Pain intensity was assessed daily through completion of diary cards from the start of the run‐in period (Day −7) to the final follow‐up visit on Day 35 in both studies. Scores for pain intensity were measured using a NPRS from 0 (no pain) to 10 (worst pain imaginable) and recorded in the diary cards. Allodynia and hyperalgesia scores were measured using a visual analogue scale (VAS) from 0 to 100 mm after applying a stimulus (brushing for allodynia, pinprick for hyperalgesia); higher scores indicated greater pain severity.

In both studies, only medications that did not interfere with the interpretation of study results and those medically indicated for the treatment of AEs were permitted during the study. Patients continued with their chronic medications if they had been stable in the previous month (or last 3 months for diabetes treatment). In both studies, permitted rescue medications were paracetamol (1000 mg orally every 6 h) and metamizole (up to 2 g orally every 8 h). If at any time the investigator considered that the patient required any other analgesic treatment in addition to the study medication or the permitted rescue medication, the patient was discontinued from the study to allow appropriate treatment.

### 2.3 Outcomes

The CPSP and PDN studies were both exploratory phase 2 studies and, therefore, no formal primary or secondary endpoints were defined. The following efficacy assessments were conducted in both studies: mean pain intensity (24‐h average and worst pain) in the previous 7 days using a NPRS; mean allodynia and hyperalgesia using a VAS; short‐form Brief Pain Inventory (SF‐BPI) (Cleeland, 2009); short‐form McGill Pain Questionnaire (SF‐MPQ) (Melzack, 1987); Patient Global Impression of Change (PGIC); use of rescue medication; and change in Neuropathic Pain Symptom Inventory (NPSI) score from baseline. Pre‐specified subgroup analyses were conducted in both studies. Safety assessments included AEs (identified from spontaneous reporting by patients and from asking patients non‐leading questions), vital signs and laboratory parameter abnormalities. A full list of outcomes and their definitions are included in the Data S1.

### 2.4 Statistical analysis

As the CPSP and PDN studies were both exploratory, sample sizes were not formally calculated, but were estimated according to clinical criteria. A total of 120 patients were planned to be enrolled in each study.

Efficacy analyses were performed in the full analysis set (FAS) and per‐protocol analysis set (PPAS) for both studies. Efficacy data reported in this manuscript are primarily based on the FAS, unless stated otherwise. The FAS comprised all randomized patients who took study medication and provided at least one valid baseline and on‐treatment observation for efficacy variables. The PPAS comprised a subset of FAS patients who had no major protocol deviations and were on treatment for ≥7 days. Safety analyses were performed in the safety analysis set, defined as all randomized patients who received at least one dose of study medication.

Time‐specific changes from baseline pain intensity scores were analysed using an analysis of variance model. Fixed effects included factors for treatment and centre, and the baseline value of the variable was used as a covariate. Ninety‐five percent confidence intervals (CIs) for the least squares (LS) mean of the treatment difference were derived for each time point. A Mantel–Haenszel chi‐square test adjusted for study centre was used to analyse the proportions of patients with reductions from baseline of ≥30% and ≥50% from baseline in the 24‐h average pain score in the previous 7 days using a NPRS, and the proportions of patients needing rescue medication.

### 2.5 Role of the funding source

Employees of the sponsor, ESTEVE Pharmaceuticals, participated in the study designs and conduct, data review and interpretation, drafting of the article and decision to submit for publication. Site management, data collection and statistical analyses were conducted by independent contract research organizations (CPSP Study: RPS Strategic Solutions, a division of PRA Health Sciences [acquired by ICON]; PDN Study: Premier Research).

## 3 RESULTS

### 3.1 Patients

**FIGURE 2:**
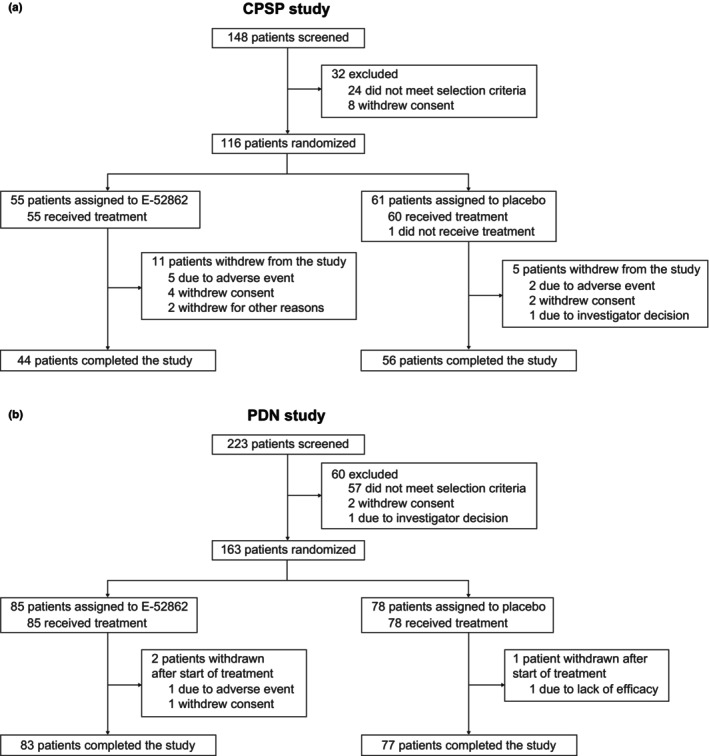
Patient disposition in the (a) CPSP Study and (b) PDN Study. CPSP, chronic postsurgical pain; PDN, painful diabetic neuropathy.

In the CPSP Study, 116 patients were included, with 55 patients randomized to E‐52862 and 61 to placebo (FAS). All patients in the E‐52862 group and 60 patients in the placebo group (98.4%) received treatment. In total, 16 patients (11 in the E‐52862 group and 5 in the placebo group) withdrew from the study due to AEs, withdrawal of consent or for other reasons (Figure 2a). In the PDN Study, 163 patients were included, with 85 patients randomized to E‐52862 and 78 to placebo (FAS). All patients in both groups received treatment. Three patients (two in the E‐52862 group and one in the placebo group) withdrew due to an AE, lack of efficacy or withdrawal of consent (Figure 2b).

Patients enrolled in the CPSP Study were younger on average than those in the PDN Study; median ages were 53 years (range: 20–81) and 61 years (range: 23–75 years), respectively (Table 1). The CPSP Study also enrolled a higher proportion of female patients (68.4%) relative to the PDN Study (51.5%). Almost all patients were white (CPSP Study: 96.5%; PDN Study: 100%). Mean average and worst pain scores at baseline were higher in the CPSP Study than in the PDN Study. Additionally, patients in both studies had high NPSI scores at baseline, although patients in the CPSP Study had higher mean baseline scores than patients in the PDN Study for NPSI total pain intensity score and all NPSI dimensional scores, with the exception of superficial spontaneous pain, and paresthesia/dysesthesia, which were similar across studies.

**TABLE 1:** Baseline demographics and clinical characteristics in the CPSP and PDN studies (full analysis set unless indicated otherwise).

	CPSP Study		PDN Study	
	E‐52862	Placebo	E‐52862	Placebo
	*n* = 55	*n* = 59	*n* = 85	*n* = 78
Median age, years (range)	51.0 (32–81)	55.0 (20–74)	61.0 (23–75)	60.5 (24–74)
Sex, *n* (%)				
Female	36 (65.5%)	42 (71.2%)	42 (49.4%)	42 (53.8%)
Male	19 (34.5%)	17 (28.8%)	43 (50.6%)	36 (46.2%)
Race, *n* (%)				
White	51 (92.7%)	59 (100%)	85 (100%)	78 (100%)
Other	4 (7.3%)	0	0	0
Mean weight, kg (SD)	78.0 (15.4)^a^	74.7 (15.6)^a^	85.8 (16.2)	87.3 (16.6)
Country of enrolment, *n* (%)				
Spain	55 (100%)	59 (100%)	16 (18.8%)	14 (17.9%)
Romania	0	0	69 (81.2%)	64 (82.1%)
Mean average NPRS score at baseline (SD)	6.2 (1.6)	6.5 (1.5)	5.3 (1.7)	5.4 (1.6)
Mean worst NPRS score at baseline (SD)	8.6 (1.2)	8.4 (1.0)	6.6 (1.5)	7.1 (1.5)
Mean NPSI absolute value at baseline (SD)				
Superficial spontaneous pain	5.0 (3.6)^b^	5.5 (3.3)^b^	5.2 (2.6)^c^	5.0 (2.9)^c^
Deep spontaneous pain	10.8 (6.3)^b^	12.4 (4.7)^b^	4.1 (2.6)^c^	4.3 (2.5)^c^
Paroxysmal pain	12.0 (4.3)^b^	11.5 (5.2)^b^	4.3 (2.8)^c^	4.3 (2.5)^c^
Evoked pain	19.6 (6.6)^b^	19.0 (6.2)^b^	4.0 (2.6)^c^	4.5 (2.4)^c^
Paresthesia/dysesthesia	12.1 (4.6)^b^	12.1 (4.6)^b^	6.0 (2.2)^c^	6.1 (2.4)^c^
Total pain intensity score	59.4 (16.1)^d^	60.6 (14.9)^d^	45.8 (19.3)^d^	47.9 (18.2)^d^
Patients who received any prior neuropathic pain treatment, *n* (%)	49 (89.1%)	56 (94.9%)	75 (88.2%)	69 (88.5%)
Prior neuropathic pain treatment reported in ≥8% of patients, *n* (%)				
Amitriptyline	3 (5.5%)	6 (10.2%)	–	–
Capsaicin	10 (18.2%)	15 (25.4%)	–	–
Dexketoprofen	5 (9.1%)	2 (3.4%)	–	–
Diclofenac	5 (9.1%)	2 (3.4%)	–	–
Duloxetine	12 (21.8%)	11 (18.6%)	–	–
Fentanyl	2 (3.6%)	5 (8.5%)	–	–
Gabapentin	9 (16.4%)	12 (20.3%)	–	–
Ibuprofen	12 (21.8%)	9 (15.3%)	–	–
Lidocaine	17 (30.9%)	20 (33.9%)	–	–
Metamizole	18 (32.7%)	20 (33.9%)	–	–
Paracetamol	24 (43.6%)	30 (50.8%)	–	–
Paracetamol, tramadol hydrochloride	6 (10.9%)	4 (6.8%)	–	–
Pregabalin	27 (49.1%)	27 (45.8%)	–	–
Tapentadol	3 (5.5%)	5 (8.5%)	–	–
Tramadol	14 (25.5%)	15 (25.4%)	–	–
Prior treatment for PDN reported in ≥8% of patients, *n* (%)				
Antidepressants, other	–	–	7 (8.2%)	1 (1.3%)
Other analgesics and antipyretics^e^	–	–	18 (21.2%)	21 (26.9%)
Various alimentary tract and metabolism products	–	–	29 (34.1%)	27 (34.6%)
Vitamin combinations	–	–	44 (51.8%)	41 (52.6%)
Vitamin B1 and B6	–	–	15 (17.6%)	11 (14.1%)
**CPSP Study‐specific characteristics**				
Mean DN4 questionnaire score (SD)	6.9 (1.5)	7.1 (1.4)	–	–
Site of surgery, *n* (%)				
Abdomen	3 (5.5%)	8 (13.6%)	–	–
Breast	2 (3.6%)	3 (5.1%)	–	–
Gynaecological	1 (1.8%)	0	–	–
Inguinal	10 (18.2%)	12 (20.3%)	–	–
Lower limb	12 (21.8%)	10 (16.9%)	–	–
Upper limb	11 (20.0%)	11 (18.6%)	–	–
Spinal	11 (20.0%)	9 (15.3%)	–	–
Thorax	5 (9.1%)	6 (10.2%)	–	–
Mean time since surgery, years (SD)	3.3 (3.7)^f^	3.9 (3.9)^f^	–	–
**PDN Study‐specific characteristics**				
Diabetes type, *n* (%)				
1	–	–	12 (14.1)	11 (14.1)
2	–	–	73 (85.9)	67 (85.9)
Mean duration of diabetes, years (SD)	–	–	13.0 (9.0)	10.3 (7.5)
Mean duration of PDN, years (SD)	–	–	2.3 (1.3)	2.2 (1.4)
Mean HbA1c level, % (SD)	–	–	7.6 (1.3)	7.3 (1.0)
Mean MNSI (part B) score (SD)	–	–	5.1 (0.9)	5.0 (1.1)

Abbreviations: CPSP, chronic postsurgical pain; DN4, Douleur Neuropathique en 4 Questions; HbA1C, glycosylated haemoglobin; MNSI, Michigan Neuropathy Screening Instrument; NPRS, numerical pain rating scale; NPSI, Neuropathic Pain Symptom Inventory; PDN, painful diabetic neuropathy; SD, standard deviation; −, not applicable/not reported.

^a^ Data are for the safety analysis set: E‐52862 (*n* = 55) and placebo (*n* = 60).

^b^ NPSI dimension score ranges: superficial spontaneous pain, 0–10; deep spontaneous pain, 0–20; paroxysmal pain, 0–20; evoked pain, 0–30; paresthesia/dysesthesia, 0–20.

^c^ NPSI dimension score ranges: superficial spontaneous pain, 0–10; deep spontaneous pain, 0–10; paroxysmal pain, 0–10; evoked pain, 0–10; paresthesia/dysesthesia, 0–10.

^d^ NPSI total pain intensity score ranges: 0–100.

^e^ Drugs included within this classification were pregabalin and gabapentin.

^f^ Data have been converted from days into years.

Key baseline characteristics were generally balanced between the E‐52862 and placebo arms in both studies. In the CPSP Study, the average and worst NPRS scores and DN4 questionnaire scores at baseline were similar in the E‐52862 and placebo groups (average NPRS: 6.2 and 6.5, respectively; worst NPRS: 8.6 and 8.4, respectively; DN4 questionnaire score: 6.9 and 7.1, respectively). Average time since surgery was similar between groups (3.3 and 3.9 years, respectively). In the PDN Study, most patients in both the E‐52862 and placebo groups were enrolled in Romania and had type 2 diabetes. Across the treatment groups, mean MNSI (part B) score was 5.0–5.1, and the duration of PDN was 2.2–2.3 years. Patients in the E‐52862 group had a longer mean duration of diabetes than those in the placebo group (13.0 and 10.3 years, respectively) and a slightly lower worst NPRS score at baseline (6.6 vs. 7.1, respectively). In both CPSP and PDN studies, most patients had received prior neuropathic pain treatment (88.2%–94.9% across groups); however, the types of medication used were different in the CPSP and PDN studies (Table 1; Tables S3 and S4).

### 3.2 Efficacy

#### 3.2.1 CPSP study

**FIGURE 3:**
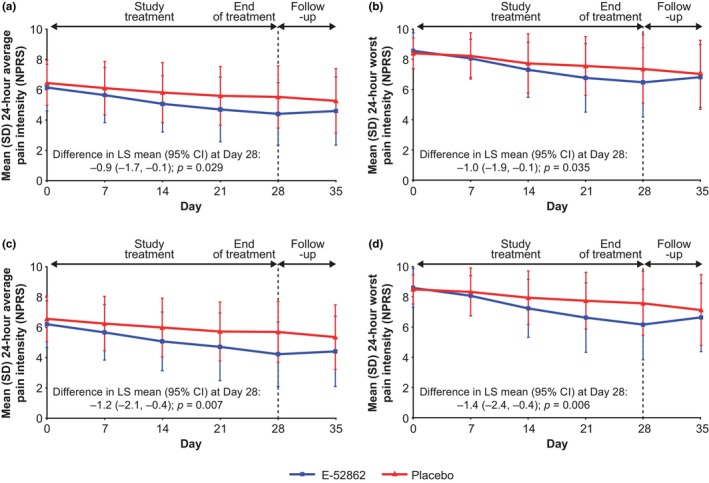
Overall mean 24‐h pain intensity by NPRS over the previous 7 days in the CPSP Study (full analysis set): (a) Overall average pain; (b) Overall worst pain; (c) Average pain in patients with non‐spinal surgery; and (d) Worst pain in patients with non‐spinal surgery. Corresponding data for patients with spinal surgery are reported in Figure S1. CI, confidence interval; CPSP, chronic postsurgical pain; LS, least squares; NPRS, numerical pain rating scale; SD, standard deviation.

In the CPSP Study, E‐52862 was associated with reductions in both 24‐h average pain intensity and worst pain intensity compared with placebo (Figure 3a, Figure 3b and Table 2). Mean average pain score reduced from 6.2 at baseline to 4.4 at Week 4 with E‐52862, and from 6.5 to 5.5 with placebo; difference in LS mean change from baseline (CFB) [95% CI]: −0.9 [−1.7, −0.1]; *p* = 0.029. Mean worst pain score reduced from 8.6 at baseline to 6.5 at Week 4 with E‐52862, and from 8.4 to 7.4 with placebo; difference in LS mean CFB [95% CI]: −1.0 [−1.9, −0.1]; *p* = 0.035). Pain scores remained below pre‐treatment values in the off‐treatment window (Days 29–36) but were not significantly different between treatment groups.

**TABLE 2:** Summary of efficacy outcomes in the CPSP Study (full analysis set unless indicated otherwise).

	E‐52862 *n* = 55	Placebo *n* = 59	Difference in least squares mean (95% CI)	*p* value	Relative risk (95% CI)
Mean change in average NPRS score in the previous 7 days from baseline to Week 4 (SD)					
All patients	−1.6 (1.9)	−0.9 (1.8)	−0.9 (−1.7, −0.1)	0.029	–
Surgery location					
Non‐spinal surgery location	*n* = 44 –1.9 (2.0)	*n* = 50 –0.8 (1.7)	–1.2 (–2.1, –0.4)	0.007	–
Spinal surgery location	*n* = 10 –0.6 (1.3)	*n* = 9 –1.2 (2.2)	–0.2 (–2.1, 1.6)	0.786	–
Pain intensity at baseline					
Moderate (≥4 to ≤6)^a^	*n* = 24 −1.2 (1.6)	*n* = 17 −0.8 (1.8)	−0.4 (−1.9, 1.1)	0.602	–
Severe (>6)^a^	*n* = 17 −2.4 (2.6)	*n* = 28 −1.3 (2.0)	−1.4 (−3.1, 0.3)	0.097	–
Prior neuropathic pain treatment					
Naïve^a^	*n* = 10 −1.5 (1.9)	*n* = 12 −0.9 (2.0)	0.8 (−2.5, 4.1)	0.602	–
Non‐naïve^a^, ^b^	*n* = 31 −1.7 (2.2)	*n* = 33 −1.2 (1.9)	−0.9 (−2.1, 0.3)	0.147	–
Time since surgery					
≤400 days^a^	*n* = 11 −1.4 (1.3)	*n* = 10 −0.6 (1.7)	0.4 (−1.7, 2.4)	0.699	–
>400 days^a^	*n* = 30 −1.8 (2.4)	*n* = 35 −1.2 (2.0)	0.1 (−1.2, 1.4)	0.890	–
Sex					
Female^a^	*n* = 36 −2.2 (2.3)	*n* = 42 −1.1 (1.8)	−1.3 (−2.6, 0.1)	0.065	–
Male^a^	*n* = 19 −0.9 (1.4)	*n* = 17 −1.2 (2.1)	0.6 (−1.3, 2.5)	0.544	–
Mean change in worst NPRS score in the previous 7 days from baseline to Week 4 (SD)					
All patients	−2.0 (2.3)	−1.0 (2.1)	−1.0 (−1.9, −0.1)	0.035	–
Surgery location					
Non‐spinal surgery location	*n* = 44 −2.3 (2.3)	*n* = 50 −0.9 (1.7)	−1.4 (−2.4, –0.4)	0.006	–
Spinal surgery location	*n* = 10 7.5 (2.0)	*n* = 9 6.3 (2.8)	0.3 (−2.0, 2.6)	0.802	–
Pain intensity at baseline					
Moderate (≥4 to ≤6)^a^	*n* = 24 −1.7 (2.0)	*n* = 17 −1.4 (2.8)	−0.1 (−2.1, 2.0)	0.962	–
Severe (>6)^a^	*n* = 17 −2.4 (3.0)	*n* = 28 −1.2 (1.9)	−1.1 (−3.0, 0.7)	0.208	–
Sex					
Female^a^	*n* = 36 −2.6 (2.7)	*n* = 42 −1.4 (2.2)	−0.7 (−2.5, 1.0)	0.400	–
Male^a^	*n* = 19 −1.0 (1.4)	*n* = 17 −1.1 (2.5)	0.7 (−1.6, 2.9)	0.524	–
Patients with a ≥30% or ≥50% reduction in average NPRS score in the previous 7 days from baseline to Week 4, *n* (%)					
≥30%	19 (34.5%)	9 (15.3%)	–	0.004	2.7 (1.3, 5.4)
≥50%	9 (16.4%)	5 (8.5%)	–	0.122	2.2 (0.8, 6.3)
Median time to onset of sustained therapeutic improvement (≥1 point reduction in mean NPRS score from baseline, with a ≥30% reduction in average NPRS score from baseline to Week 4), days (95% CI)	18.0 (8.0, NC)	28.0 (8.0, NC)	–	–	–
Mean SF‐MPQ change from baseline to Week 4 (SD)					
Sensory total score	−4.6 (9.2)	−5.2 (8.8)	0.9 (−2.3, 4.2)	0.579	–
Affective total score	−1.8 (3.8)	−2.2 (3.7)	0.4 (−0.9, 1.6)	0.589	–
Overall total score	−6.4 (12.2)	−7.3 (11.6)	1.2 (−3.1, 5.6)	0.574	–
Visual analogue scale, cm	−2.0 (3.1)	−1.4 (2.6)	−0.8 (1.8, 0.2)	0.135	–
Mean SF‐BPI change from baseline to Week 4 (SD)					
*Items of interest for CPSP Study*					
Current pain severity score	−2.1 (3.1)	−1.6 (2.8)	−0.9 (−1.9, 0.2)	0.107	–
Pain interference score	−2.2 (3.2)	−1.7 (3.3)	−0.5 (−1.7, 0.7)	0.437	–
Mean NPSI change from baseline to Week 4 (SD)					
Superficial spontaneous pain Deep spontaneous pain Paroxysmal pain Evoked pain Paresthesia/dysesthesia Total pain intensity score	−1.6 (3.6) −3.0 (7.7) −3.7 (6.8) −6.0 (8.9) −4.9 (6.9) −19.7 (28.4)	−1.8 (3.5) −2.3 (6.9) −3.2 (6.7) −3.7 (7.6) −4.0 (5.4) −14.5 (23.7)	−0.1 (−1.2, 1.1) −2.6 (−4.9, −0.3) −0.3 (−2.7, 2.1) −2.7 (−5.7, 0.3) −1.2 (−3.4, 1.1) −8.7 (−18.5, 1.1)	0.872 0.027 0.781 0.080 0.301 0.083	– – – – – –
Patient global impression of change at Week 4, %				0.311	
Patients in “very much improved” or “much improved” categories	20 (37.0)	13 (23.2)	–		–
Patients in five remaining categories from “minimally improved” to “very much worse”	34 (63.0)	43 (76.8)	–		–
Patients receiving rescue medication, *n* (%)^a^	27 (65.9)	38 (82.6)	–	0.190	0.9 (0.7, 1.1)

Abbreviations: CI, confidence interval; CPSP, chronic postsurgical pain; NC, not calculable; NPRS, numerical pain rating scale; NPSI, Neuropathic Pain Symptom Inventory; SD, standard deviation; SF‐BPI, short‐form Brief Pain Inventory; SF‐MPQ, short‐form McGill Pain Questionnaire; −, not applicable/not reported.

^a^ Data are for the per‐protocol analysis set; E‐52862 (*n* = 41) and placebo (*n* = 46). Baseline NPRS score was missing in 1 patient in the placebo group, therefore subgroup analyses data are shown for *n* = 45 patients in the placebo group of the per‐protocol analysis set. Since this was a phase 2 study, some exploratory analyses, such as the subgroup analyses, were performed on the per‐protocol population.

^b^ Non‐naïve patients were those with any prior treatment with amitriptyline, capsaicin, duloxetine, gabapentin, lidocaine, opioids, or pregabalin.

A clinically significant response to E‐52862 was observed in the subgroup of patients with chronic pain following non‐spinal surgery, with statistically significant differences in the LS mean CFB in average pain (−1.2 [−2.1, −0.4]; *p* = 0.007) and worst pain (−1.4 [−2.4, −0.4]; *p* = 0.006) at Week 4 for the comparison of E‐52862 vs. placebo (Figure 3c and Figure 3d). In the subgroup of patients with pain following spinal surgery, there was no significant between‐group difference in the LS mean CFB for average or worst pain. Further pre‐specified subgroup analyses by pain intensity at baseline, prior neuropathic pain treatment status, time since surgery, and sex are reported in Table 2, and by age and pain localization in Table S5.

The proportion of patients achieving at least a 30% or 50% reduction in mean pain intensity from baseline to Week 4 with E‐52862 was approximately double compared with that for placebo: 34.5% vs. 15.3% for ≥30% reduction (*p* = 0.004) and 16.4% vs. 8.5% for ≥50% reduction (*p =* 0.122), respectively. Mean CFB to Week 4 in SF‐MPQ scores were similar in the E‐52862 and placebo groups. Mean pain reduction from baseline to Week 4 measured by the SF‐BPI was numerically greater with E‐52862 vs. placebo (current pain severity: *p* = 0.107; pain interference: *p* = 0.437). The mean CFB to Week 4 in NPSI total pain intensity score was numerically greater for E‐52862 than for placebo (LS mean difference, −8.7 [−18.5, 1.1], *p* = 0.083). For the PGIC, a numerically higher proportion of patients selected the “very much improved” or “much improved” categories for global impression of change at Week 4 with E‐52862 (37.0%) vs. the placebo group (23.2%). Overall, there was no significant difference in PGIC response rates between E‐52862 and placebo (*p* = 0.311, Wilcoxon rank sum) (Table S6). A numerically greater proportion of patients in the placebo group (82.6%) compared with the E‐52862 group (65.9%) received rescue medication (*p* = 0.190; PPAS data) across all on‐treatment study visits and the off‐treatment visit at Day 35. Mean CFB to Days 7, 14, 21 and 28 in allodynia and hyperalgesia scores are shown in Table S7. Mean NPSI domain and total pain intensity scores at Days 7, 14, 21, 28 and 35 are shown in Figure S2.

#### 3.2.2 PDN study

**FIGURE 4:**
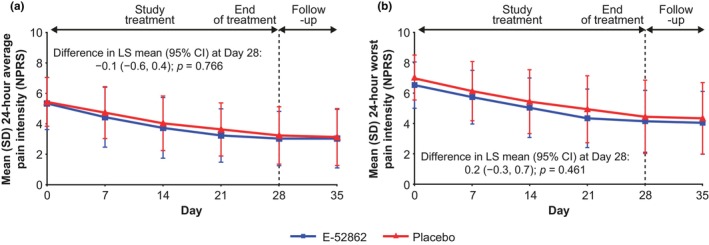
Mean 24‐h average (a) and worst (b) pain intensity by NPRS over the previous 7 days in the PDN Study (full analysis set). CI, confidence interval; LS, least squares; NPRS, numerical pain rating scale; PDN, painful diabetic neuropathy; SD, standard deviation.

In the PDN Study, mean values for 24‐h average and worst pain intensity over the previous 7 days were similar with E‐52862 and placebo and showed a constant reduction between baseline and Week 4 (Figure 4a,b). Mean average pain score was 5.3 at baseline and 3.0 at Week 4 with E‐52862; and 5.4 at baseline and 3.2 at Week 4 with placebo. Mean worst pain score was 6.6 at baseline and 4.2 at Week 4 with E‐52862; and 7.1 at baseline and 4.5 at Week 4 with placebo. No significant difference was observed between groups for the CFB to Week 4 in average pain intensity (difference in LS mean CFB [95% CI]: −0.1 [−0.6, 0.4]; *p* = 0.766) or worst pain intensity (difference in LS mean CFB [95% CI]: 0.2 [−0.3, 0.7]; *p* = 0.461) (Table 3). In subgroup analyses performed by baseline pain intensity, and by sex (Table 3), age, and HbA1C levels (Table S8), no significant differences in average or worst pain at Week 4 were found between groups.

**TABLE 3:** Summary of efficacy outcomes in the PDN Study (full analysis set).

	E‐52862 *n* = 85	Placebo *n* = 78	Difference in least squares mean (95% CI)	*p* value	Adjusted odds ratio (95% CI)
Mean change in average NPRS score in the previous 7 days from baseline to Week 4 (SD)					
All patients	−2.2 (1.8)	–2.1 (1.8)	−0.1 (−0.6, 0.4)	0.766	–
Pain intensity at baseline					
Moderate (>4 to <6)	*n* = 43^a^ −2.1 (1.7)	*n =* 46^a^ −2.0 (1.6)	−0.0 (−0.7, 0.7)	0.993	–
Severe (≥6)	*n =* 24^a^ −3.2 (1.8)	*n =* 20^a^ −3.2 (2.2)	−0.1 (−1.4, 1.2)	0.925	–
Sex					
Female	*n =* 42 −2.5 (1.8)	*n =* 42 −2.3 (1.9)	−0.3 (−1.0, 0.4)	0.366	–
Male	*n =* 43 −1.9 (1.7)	*n =* 36 −1.8 (1.7)	0.1 (−0.6, 0.8)	0.787	–
Mean change in worst NPRS score in the previous 7 days from baseline to Week 4 (SD)					
All patients	−2.3 (1.8)	−2.6 (1.9)	0.2 (−0.3, 0.7)	0.461	–
Pain intensity at baseline					
Moderate (>4 to <6)	*n =* 43^a^ −2.4 (1.5)	*n =* 46^a^ −2.6 (1.8)	0.2 (−0.4, 0.9)	0.472	–
Severe (≥6)	*n =* 24^a^ −2.8 (2.2)	*n =* 20^a^ −3.1 (2.4)	0.3 (−1.0, 1.7)	0.630	–
Sex					
Female	*n =* 42 −2.7 (1.8)	*n =* 42 −2.7 (2.0)	−0.2 (−1.0, 0.6)	0.617	–
Male	*n =* 43 −2.0 (1.7)	*n =* 36 −2.3 (1.9)	0.5 (−0.3, 1.2)	0.197	–
Patients with a ≥30% or ≥50% reduction in average NPRS score in the previous 7 days from baseline to Week 4, *n* (%)					
≥30%	53 (62.4%)	46 (59.0%)	–	0.856	1.1 (0.5, 2.1)
≥50%	33 (38.8%)	27 (34.6%)	–	0.636	1.2 (0.6, 2.4)
Median time to onset of sustained therapeutic improvement (≥1 point reduction in mean NPRS score from baseline, with a ≥30% reduction in average NPRS score from baseline to Week 4), days (95% CI)	20.0 (14.0, 27.0)	20.0 (14.0, 31.0)	–	–	–
Mean SF‐MPQ change from baseline to Week 4 (SD)					
Sensory score	−3.9 (5.2)	−5.1 (6.1)	1.2 (−0.3, 2.6)	0.106	–
Affective score	−1.0 (2.2)	−1.6 (2.2)	0.5 (−0.1, 1.1)	0.084	–
Total score	−4.8 (6.7)	−6.8 (7.5)	1.9 (0.1, 3.7)	0.042	–
Mean SF‐BPI change from baseline to Week 4 (SD)					
*Items of interest for PDN Study*					
Current pain severity score	−1.7 (2.1)	−2.2 (2.1)	0.2 (−0.3, 0.7)	0.478	–
Pain interference score	−1.6 (1.5)	−1.9 (1.8)	0.3 (−0.1, 0.7)	0.112	–
Mean NPSI change from baseline to Week 4 (SD)					
Superficial spontaneous pain Deep spontaneous pain Paroxysmal pain Evoked pain Paresthesia/dysesthesia Total pain intensity score	−1.8 (2.5) −1.6 (2.1) −1.7 (2.4) −1.4 (2.0) −2.1 (2.3) −16.5 (17.0)	−1.9 (2.6) −2.0 (2.3) −1.8 (2.3) −1.9 (2.1) −2.6 (2.2) −20.4 (16.9)	0.2 (−0.3, 0.8) 0.2 (−0.3, 0.6) −0.0 (−0.6, 0.5) 0.3 (−0.3, 0.8) 0.4 (−0.1, 0.9) 1.7 (−2.5, 6.0)	0.400 0.465 0.879 0.322 0.153 0.422	– – – – – –
Patient global impression of change at Week 4, *n* (%)				0.716	
Patients in “very much improved” or “much improved” categories	44 (52.4 %)	33 (43.4 %)	–		–
Patients in five remaining categories from “minimally improved” to “very much worse”	40 (47.6 %)	42 (55.3 %)	–		–
Patients receiving rescue medication, *n* (%)	11 (12.9%)	8 (10.3%)	–	0.642	0.7 (0.2, 2.6)

Abbreviations: CI, confidence interval; NPRS, numerical pain rating scale; NPSI, Neuropathic Pain Symptom Inventory; PDN, painful diabetic neuropathy; SD, standard deviation; SF‐BPI, short‐form Brief Pain Inventory; SF‐MPQ, short‐form McGill Pain Questionnaire; −, not applicable/not reported.

^a^ Data from the subgroup of patients with mild baseline pain severity (E52862 *n* = 18; placebo *n* = 12) are not included here.

The proportion of patients with at least a 30% or 50% reduction in average pain score from baseline to Week 4 was numerically higher with E‐52862 vs. placebo: 62.4% vs. 59.0% for ≥30% reduction (*p* = 0.856) and 38.8% vs. 34.6% for ≥50% reduction (*p* = 0.636) respectively (Table 3). Mean reductions from baseline to Week 4 in SF‐MPQ scores appeared greater with placebo than E‐52862. Analysis of SF‐BPI items 5 (pain on average) and 3 (pain at its worst) showed comparable time profiles to NPRS‐measured average and worst pain, respectively (data not shown), and similar mean CFB to Week 4 in the two treatment groups (current pain severity: *p* = 0.478; pain interference: *p* = 0.112). For the PGIC, a numerically higher proportion of patients selected the “very much improved” or “much improved” categories for global impression of change at Week 4 with E‐52862 (52.4%) vs. placebo (43.4%). There was no difference in CFB to Week 4 in NPSI total pain intensity score between E‐52862 and placebo (*p* = 0.422). Overall, there was no significant difference in PGIC response rates between E‐52862 and placebo (*p* = 0.716, Wilcoxon rank sum) (Table S6). The proportion of patients who needed rescue medication was similar in the two treatment groups (12.9% with E‐52862 vs. 10.3% with placebo; *p* = 0.642). Mean CFB to Days 7, 14, 21 and 28 in allodynia and hyperalgesia scores are shown in Table S9. Mean NPSI domain and total pain intensity scores at Days 7, 14, 21, 28 and 35 are shown in Figure S3.

### 3.3 Safety

In the CPSP Study, 50 patients (90.9%) in the E‐52862 group and 46 (76.7%) patients in the placebo group experienced at least one treatment‐emergent adverse event (TEAE) (Table 4). The most commonly reported TEAEs were dizziness, headache, nausea, upper abdominal pain, and vomiting. Most TEAEs were of mild or moderate severity in both treatment groups. More patients had TEAEs that were assessed as related to study treatment in the E‐52862 group (65.5%) compared with the placebo group (38.3%). TEAEs led to study discontinuation in 5 patients (9.1%) in the E‐52862 group and were considered related to study treatment in 4/5 patients: nausea and sense of oppression; nausea, headache, upper abdominal pain and paraesthesia; vertigo; and depression. TEAEs led to discontinuation in 2 patients (3.3%) in the placebo group and were considered related to study treatment in both patients: vomiting and upper abdominal pain.

**TABLE 4:** Summary of TEAEs in the CPSP and PDN studies (safety analysis set).

	CPSP Study		PDN Study	
	E‐52862 *n* = 55	Placebo*n* = 60	E‐52862 *n* = 85	Placebo *n* = 78
Any TEAE, *n* (%)	50 (90.9%)	46 (76.7%)	29 (34.1%)	21 (26.9%)
Mild	43 (78.2%)	41 (68.3%)	22 (25.9%)	13 (16.7%)
Moderate	25 (45.5%)	20 (33.3%)	6 (7.1%)	8 (10.3%)
Severe	12 (21.8%)	5 (8.3%)	1 (1.2%)	0
Any treatment‐related TEAE, *n* (%)	36 (65.5%)	23 (38.3%)	11 (12.9%)	8 (10.3%)
Any TEAE leading to study discontinuation, *n* (%)	5 (9.1%)	2 (3.3%)	1 (1.2%)	0
Any serious TEAEs, *n* (%)	3 (5.5%)	4 (6.7%)	0	0
Deaths, *n* (%)	0	0	0	0
Most common TEAEs by preferred term (occurring in ≥5% of patients in any group in either study), *n* (%)^a^				
Dizziness	24 (43.6%)	15 (25.0%)	7 (8.2%)	5 (6.4%)
Nausea	18 (32.7%)	4 (6.7%)	8 (9.4%)	0
Headache	17 (30.9%)	13 (21.7%)	3 (3.5%)	5 (6.4%)
Upper abdominal pain	14 (25.5%)	7 (11.7%)	2 (2.4%)	1 (1.3%)
Vomiting	7 (12.7%)	1 (1.7%)	3 (3.5%)	0
Anxiety	6 (10.9%)	2 (3.3%)	0	5 (6.4%)
Dyspepsia	6 (10.9%)	1 (1.7%)	2 (2.4%)	1 (1.3%)
Insomnia	6 (10.9%)	4 (6.7%)	0	1 (1.3%)
Abdominal discomfort	6 (10.9%)	0	0	0
Arthralgia	5 (9.1%)	4 (6.7%)	0	1 (1.3%)
Dry mouth	5 (9.1%)	1 (1.7%)	1 (1.2%)	0
Back pain	4 (7.3%)	2 (3.3%)	1 (1.2%)	2 (2.6%)
Hyperhidrosis	4 (7.3%)	0	0	0
Pain in extremity	4 (7.3%)	5 (8.3%)	3 (3.5%)	1 (1.3%)
Nasopharyngitis	4 (7.3%)	1 (1.7%)	2 (2.4%)	1 (1.3%)
Constipation	4 (7.3%)	0	3 (3.5%)	0
Malaise	3 (5.5%)	3 (5.0%)	2 (2.4%)	1 (1.3%)
Flatulence	3 (5.5%)	0	0	0
Neck pain	3 (5.5%)	3 (5.0%)	0	2 (2.6%)
Somnolence	3 (5.5%)	2 (3.3%)	0	2 (2.6%)
Diarrhoea	3 (5.5%)	1 (1.7%)	0	0
Muscle spasms	2 (3.6%)	5 (8.3%)	0	0
Fatigue	2 (3.6%)	4 (6.7%)	2 (2.4%)	2 (2.6%)
Asthenia	2 (3.6%)	3 (5.0%)	1 (1.2%)	0
Toothache	1 (1.8%)	3 (5.0%)	0	0
Abdominal pain	1 (1.8%)	3 (5.0%)	1 (1.2%)	1 (1.3%)
Limb discomfort	0	3 (5.0%)	0	0

Abbreviations: TEAE, treatment‐emergent adverse event.

^a^ Data are ordered by decreasing incidence in the E‐52862 group in the CPSP Study.

In the PDN Study, 29 patients (34.1%) in the E‐52862 group and 21 patients (26.9%) in the placebo group experienced at least one TEAE (Table 4). The most commonly reported TEAEs were dizziness, nausea, headache, and anxiety. Most TEAEs were mild in severity and were not considered related to E‐52862. One patient in the E‐52862 group had a TEAE leading to study discontinuation (urinary tract infection), which was assessed as not related to study treatment.

There were no deaths in either study, and serious TEAEs were only reported in the CPSP Study; there were no serious TEAEs in the PDN Study. In the CPSP Study, serious TEAEs were reported by 3 patients (5.5%) in the E‐52862 group and 4 patients (6.7%) in the placebo group, and all had resolved at the time of follow‐up. In the E‐52862 group, the patients with serious TEAEs were: one patient with increased blood creatinine, potassium and urea (all of which were assessed as related to study treatment); one patient with infection (assessed as unrelated to study treatment and resulting in discontinuation from the study); and one patient with procedural pain (assessed as unrelated to study treatment). In the placebo group, the patients with serious TEAEs were: one patient with increased alanine aminotransferase and gamma‐glutamyl transferase (both assessed as unrelated to study treatment); one patient with vomiting (assessed as related to study treatment and resulting in discontinuation from the study); one patient with gastrointestinal obstruction (assessed as unrelated to study treatment); and one patient with abdominal pain (assessed as related to study treatment and resulting in discontinuation from the study).

## 4 DISCUSSION

We report analyses of two phase 2, randomized, proof‐of‐concept studies of E‐52862, a novel, selective S1RA, in patients with moderate‐to‐severe CPSP and PDN.

In patients with CPSP, E‐52862 demonstrated greater reductions from baseline in average and worst 24‐h pain compared with placebo. While the magnitude of pain relief based on changes in average pain intensity were small, the improvement in 30% responder rates with E‐52862 vs. placebo, combined with the difficult‐to‐treat population (patients with moderate‐to‐severe chronic pain, most of whom had shown an unsatisfactory response to other drugs), suggest a clinically relevant treatment effect. Clinical relevance is further emphasized by the scarcity of evidence for CPSP treatment (Kim et al., 2023); most studies in peripheral neuropathic pain have been conducted in post‐herpetic neuralgia or PDN (Attal et al., 2023) and efficacy is typically extrapolated in other peripheral neuropathic pain conditions. Moreover, other peripheral neuropathic pain trials have demonstrated smaller pain score improvements despite longer treatment duration (Aiyer et al., 2017; Markman et al., 2018; Shetty et al., 2024).

Results of the CPSP Study subgroup analyses numerically mirrored the main analysis, although treatment differences were not all statistically significant, perhaps due to small sample sizes and short study duration. Notably, average and worst pain were reduced in patients with non‐spinal surgery; a potentially more generalizable result than the spinal surgery subgroup, where the poorer response is likely due to the mixed, rather than solely neuropathic, pain associated with spinal surgery (Bajwa & Haldar, 2015) and potential confounding effects of failed back surgery syndrome (Sebaaly et al., 2018). E‐52862 appeared to improve pain scores regardless of baseline pain intensity (2.4‐point reduction from baseline pain scores at Week 4 in patients with severe baseline pain), prior neuropathic pain treatment, or time since surgery, but these observations did not reach statistical significance. This is clinically meaningful for chronic pain relief and the refractory population, where the mean time since surgery was 3.6 years and around 75% of patients had been treated unsuccessfully with approved or recommended treatments for peripheral neuropathic pain (e.g. pregabalin, gabapentin, duloxetine, capsaicin, amitriptyline, lidocaine, or opioids). Furthermore, there are few effective treatments for CPSP, with limited clinical evidence for most interventions (Attal et al., 2023; Rosenberger & Pogatzki‐Zahn, 2022).

In patients with moderate‐to‐severe PDN, there was a similar reduction in 24‐h average and worst pain intensity from baseline, as well as in NPSI score, in both the E‐52862 and placebo groups. Although a placebo effect is not unusual for clinical pain trials, particularly PDN (Dworkin et al., 2010), the magnitude of the placebo response was particularly high in the context of other randomized controlled trials (Colloca, 2019; Frisaldi et al., 2017; Frisaldi et al., 2023; Vase & Wartolowska, 2019). Outside of study‐related elements (e.g. study duration, choice of naïve patients), several factors may contribute to placebo responses in pain studies, including genetic, psychological and biological characteristics (Vase & Wartolowska, 2019). Face‐to‐face study visits (Vase et al., 2015), and the older average age of patients in the PDN Study compared with the CPSP Study, may have been contributing factors to the observed effect. Patients' expectation of a therapeutic intervention and its efficacy are key drivers (Colloca, 2019; Frisaldi et al., 2017; Vase & Wartolowska, 2019). Development of more precise placebo controls and blinding procedures, and assessment of patients' expectations in routine practice, are needed to control this effect (Frisaldi et al., 2017; Vase & Wartolowska, 2019). Methodological factors such as these, and more general regional differences in the conduct of the CPSP and PDN studies reported here, may have contributed to the observed difference in treatment effects between the two studies. There were also differences in prior pain medication exposure; use of medications with a neuropathic pain indication or endorsement by clinical practice guidelines was marginal in the PDN Study compared to the CPSP Study.

Development of neuropathic pain interventions acting on new targets is challenging; many monotherapies have only moderate efficacy and/or limiting side effects (Attal et al., 2023; Cavalli et al., 2019; Chincholkar, 2020). For example, despite promising outcomes in animal models, the sodium channel blocker lacosamide did not demonstrate improved pain relief vs. placebo in patients with peripheral neuropathic pain (Carmland et al., 2024). In addition, recent phase 2b studies of the selective angiotensin II type 2 receptor antagonist EMA‐401 in patients with post‐herpetic neuralgia (EMPHENE) and PDN (EMPADINE) showed a greater numerical reduction in NPRS score with EMA‐401 vs. placebo (Rice et al., 2021). Alongside improving monotherapy benefit:risk profiles, multimodal treatment approaches that combine different mechanisms of action with the goal of achieving better pain relief through additive effects using lower drug doses could be an alternative approach (Bates et al., 2019).

It is possible that larger treatment effects may have been observed in both studies reported here if higher doses or a longer duration of active treatment were investigated; this would be consistent with preclinical pharmacokinetic and pharmacodynamic modelling (Abadias et al., 2013; Romero et al., 2012; Täubel et al., 2015).

E‐52862 had an acceptable safety profile in CPSP and PDN. Considerably fewer TEAEs were reported in the PDN Study compared with the CPSP Study, possibly due to differences in the study centre locations and potential regional variations in TEAE reporting (Guimarães et al., 2017; Keebler et al., 2020). The higher mean weight of patients in the PDN Study compared with the CPSP Study may also have contributed. Except for one patient who reported serious TEAEs of increased blood creatinine, potassium and urea (all considered related to E‐52862), serious TEAEs in the CPSP Study were in accordance with the known safety profile of E‐52862. No serious TEAEs were reported in the PDN Study. This is a clear distinction from classical first‐line drugs for peripheral neuropathic pain.

Results of the CPSP and PDN studies are complemented by a previous exploratory, randomized, double‐blind, placebo‐controlled, phase 2 trial of E‐52862 in patients with postherpetic neuralgia (SIGM‐203) (EU Clinical Trials Register, 2013). Numerically greater reductions in average and worst pain were seen with E‐52862 400 mg QD vs. placebo over 28 days; however, the study was terminated early due to poor recruitment, and no statistical comparisons were performed (EU Clinical Trials Register, 2013).

Patient selection is key for conclusive assessment of study treatments against placebo in neuropathic pain trials. Recent guidelines from the International Association for the Study of Pain emphasize the importance of choosing the correct patient screening questionnaires, and provide a strong recommendation for the use of DN4 and the Leeds Assessment of Neuropathic Symptoms and Signs (LANSS) (Truini et al., 2023). Our use of DN4 for patient selection is, therefore, a validated approach and strength of the CPSP Study. Similarly, the difference in NPSI dimensions and total scores between patients in the CPSP and PDN studies and the lack of sensitivity in the NPSI total pain score at the end of treatment in our studies, similar to previously published studies (Rice et al., 2021), confirms the importance of sensory phenotyping at baseline in clinical trials of neuropathic pain conditions. Some flawed studies in neuropathic pain have shown significant effects on specific symptoms, suggesting potential influence of different neuropathic pain phenotypes. The existence of multiple neuropathic pain phenotypes is now established (Bouhassira et al., 2021; van Velzen et al., 2020), and inadequate classification of these phenotypes has been suggested as one reason for failure within neuropathic pain trials (Baron et al., 2012; Baron et al., 2023; Bouhassira & Attal, 2019; Marchevsky et al., 2021). Although the European Medicines Agency endorses patient stratification based on sensory phenotype in clinical trials, this approach is untested in large‐scale trials (Attal et al., 2023; Bouhassira & Attal, 2023). MNSI, a validated tool for diagnosing distal peripheral diabetic neuropathy, was used in the PDN Study and, once diagnosed and if painful, the intensity of pain was assessed using a NPRS scale. A specific neuropathic pain assessment scale (e.g. DN4, LANSS) was not used, which is a potential limitation of the PDN Study.

Both the CPSP and PDN studies were proof‐of‐concept studies. Limitations include the short study treatment duration, relatively small number of patients (who were predominantly White), and the previously discussed high placebo response rates in the PDN Study, which may not have allowed for differentiation from E‐52862. A substantial proportion of patients included in the CPSP Study were a refractory, difficult‐to‐treat population and although this may limit the generalization of the study results, this also increases the potential clinical relevance of these findings given limited effective treatment options for these patients. In this report, we have presented the analyses previously performed for these two proof‐of‐concept trials; however, further analyses exploring the effects of modifiers of response are warranted. Of particular interest is a post‐hoc analysis of how response to E‐52862 may differ by specific sensory phenotype, as defined by NPSI dimensional scores at baseline, using the algorithm developed by Bouhassira and colleagues (Bouhassira et al., 2021). The relatively high frequency of TEAEs observed in the CPSP Study could have resulted in guessing of a patient's treatment assignment and consequently, possible bias in assessing some outcomes. The study design mitigated against this by keeping all patients, investigators and personnel involved in the study blinded to the identity of treatment received by each patient, until the clean study database was locked for analysis. The most reported TEAE in the CPSP Study was dizziness in both treatment groups, suggesting the mitigation measures were successful in minimizing potential bias.

The efficacy, safety and tolerability evidence for E‐52862 tends to validate the S1RA mode of action, supporting its further development in peripheral neuropathic pain. Next‐generation S1RA compounds are in the preclinical phase of development and anticipated to enter early‐phase clinical development soon. To progress the clinical development programme for E‐52862 in chronic neuropathic pain, chronic preclinical toxicology data were required in two animal species. The rhesus monkey was used because it has similar exposure and metabolic profiles to humans. However, a suitable second species could not be identified due to the very low exposures of E‐52862 in other animal models compared with the exposure in humans. Moreover, the low safety margin (at the established no‐observed‐adverse‐effect level) in the longest chronic rhesus monkey toxicity study (39 weeks) would not allow for a dose increase based on the predictions from the pharmacokinetic/pharmacodynamic models in other species. Therefore, the clinical development programme for E‐52862 did not progress to longer duration confirmatory studies in chronic treatments.

## 5 CONCLUSIONS

E‐52862 provided superior relief of CPSP compared with placebo after 4 weeks of treatment in patients with moderate‐to‐severe CPSP. For patients with PDN, E‐52862 provided reductions in pain intensity; however, high placebo response rates may not have allowed for differentiation of active treatment versus placebo. E‐52862 had acceptable tolerability in both patient populations. These studies collectively support continued exploration of the S1RA mode of action in chronic peripheral neuropathic pain.

## AUTHOR CONTRIBUTIONS

RG, JC, AM, MS, and AV contributed to the conception or design of the studies. RG, FJM, AM‐P, and FNR contributed to data collection. All authors contributed to data analysis and/or interpretation. All authors drafted, edited, or reviewed drafts of the manuscript, approved the final version, and agree to be accountable for the accuracy and integrity of the work.

## FUNDING INFORMATION

These studies were funded by ESTEVE Pharmaceuticals S.A. (Barcelona, Spain). Medical writing support was funded by ESTEVE Pharmaceuticals S.A. Scientists employed by the funder participated in the design and conduct of the studies, data review and interpretation, decision to publish and drafting of the article. All authors had full access to all study data and had final responsibility for the decision to submit for publication.

## CONFLICT OF INTEREST STATEMENT

RG, VM, FJM, AM‐P, FN‐R and LC report no conflicts of interest. JC, AM, MS and AV are employees of ESTEVE Pharmaceuticals S.A. (Barcelona, Spain). DB reports consulting fees from Bayer AG, ESTEVE Pharmaceuticals S.A. and Grünenthal Ltd., and honoraria from Grünenthal Ltd.

## Supporting information


Data S1.

## Data Availability

ESTEVE Pharmaceuticals S.A. (Barcelona, Spain) will consider requests for de‐identified patient‐level data and supporting study documents from qualified external researchers. Approval of requests will be at the discretion of ESTEVE Pharmaceuticals S.A. and will depend on the scientific merit of the proposed research and intended use of the data. If approval is granted, a data sharing agreement must be signed, and access to data will be provided only if ESTEVE Pharmaceuticals S.A. has legal authority to provide the data and there are no contradictory requirements relating to regulatory filings or reviews. Proposals should be sent to esteve@esteve.com.
