# Supplementary material for: E‐52862—A selective sigma‐1 receptor antagonist, in peripheral neuropathic pain: Two randomized, double‐blind, phase 2 studies in patients with chronic postsurgical pain and painful diabetic neuropathy
Source: Eur J Pain. 2024 Dec 4;29(1):e4755. doi: 10.1002/ejp.4755 (PMC11616472; doi:10.1002/ejp.4755)
Supplement: Supplementary file 1 — Data S1. [file EJP-29-0-s001.pdf]

SUPPLEMENTARY MATERIAL

E-52862—A selective sigma-1 receptor antagonist, in peripheral neuropathic pain: Two randomized, double-blind, phase 2 studies in patients with chronic postsurgical pain and painful diabetic neuropathy

R. Gálvez, V. Mayoral, J. Cebrecos, F. J. Medel, A. Morte, M. Sust, A. Vaqué, A. Montes-Pérez, F. Neira-Reina, L. Cánovas, C. Margarit, D. Bouhassira

CONTENTS

SUPPLEMENTARY METHODS 3

S1. ELIGIBILITY CRITERIA 3

CPSP Study 3

PDN Study 4

S2. FULL LIST OF STUDY OUTCOMES AND DEFINITIONS FOR THE CPSP AND PDN STUDIES 6

Efficacy endpoints: 6

Safety endpoints: 7

S3. RANDOMIZATION 8

SUPPLEMENTARY TABLES AND FIGURES 9

TABLE S1 CPSP Study investigators (SIGM-205). 9

TABLE S2 PDN Study investigators (SIGM-204). 10

TABLE S3 Prior neuropathic pain medications for patients in the CPSP Study. 12

TABLE S4 Prior medications for patients in the PDN Study. 15

TABLE S5 Efficacy outcomes of subgroup analyses in the CPSP Study (per-protocol analysis set). 17

TABLE S6 Patient global impression of change at Week 4 in the CPSP Study and the PDN Study (full analysis set).. 19

TABLE S7 Change from baseline in allodynia and hyperalgesia VAS scores in the CPSP Study (full analysis set). 20

TABLE S8 Efficacy outcomes of subgroup analyses in the PDN Study (full analysis set)... 21

FIGURE S1 Mean 24-hour average (a) and worst (a) pain intensity by NPRS over the previous 7 days in the CPSP Study in patients with spinal surgery pain (full analysis set)... 25

FIGURE S2 Mean NPSI domain and total intensity scores in the previous 7 days: superficial spontaneous pain (a), deep spontaneous pain (b), paroxysmal pain (c), evoked pain (d), paresthesia/dysesthesia (e), and NPSI total pain intensity score (f) in the CPSP Study (full analysis set). 26

FIGURE S3 Mean NPSI domain and total intensity scores in the previous 7 days: superficial spontaneous pain (a), deep spontaneous pain (b), paroxysmal pain (c), evoked pain (d), paresthesia/dysesthesia (e), and NPSI total pain intensity score (f) in the PDN Study (full analysis set). 27

SUPPLEMENTARY METHODS

S1. ELIGIBILITY CRITERIA

CPSP Study

Inclusion criteria

Signed consent before study entry; willingness to understand and comply with protocol requirements; ≥18 years old; had undergone surgery ≥3 months before inclusion in the study; Douleur Neuropathique en 4 Questions score of ≥4; pain/allodynia and/or hyperalgesia location consistent with surgical area; moderate-to-severe pain intensity at screening and during the 7-day run-in period before Visit 2 (Day –1), defined as a mean score ≥4 on the numerical pain rating scale (NPRS); male and female individuals were eligible – female patients of childbearing age were required to use an acceptable form of contraception during the study and up until 1 month after administration of the last dose of study medication; male patients were required to use an acceptable form of contraception if their female partner(s) was (were) pregnant or could become pregnant from the time of the first administration of the study medication until 3 months following administration of the last dose of study medication.

Exclusion criteria

Pregnant or nursing; severe pain related to other nonsurgical causes; exposure to drugs known to cause neuropathy in the 30 days before receiving study medication; major psychiatric disorder; serious/unstable cardiovascular disease that could compromise participation or cause hospitalization; second/third degree atrioventricular blockade not corrected with a pacemaker or any clinically significant abnormality in the 12-lead electrocardiogram as determined by the investigator; taking specific drugs/drug classes that could not be discontinued – if discontinued, a minimum washout period of at least 5 × the half-life was required (benzodiazepines, skeletal muscle relaxants, orally administered steroids, centrally acting analgesics, opiates, topical lidocaine or capsaicin, non-steroidal anti-inflammatory drugs, anticonvulsants, tricyclic antidepressants, selective serotonin reuptake inhibitor or

serotonin and norepinephrine reuptake inhibitors, N-methyl-D-aspartate antagonists, thalidomide, nitrous oxide, Ca2+/magnesium 2+ ion infusions); treated with a drug without regulatory approval for any indication in the 30 days before study entry; history of drug abuse/dependence within 1 year before the study; history of active serious medical conditions that could compromise safety/interfere with study assessments; history of drug abuse or dependence; history of severe gastroparesis/gastric bypass surgery, positive hepatitis B or C serology, or positive HIV test/known infection; previous neurolytic/neurosurgical treatment for neuropathic pain; active malignancy or injected anaesthetics/steroid use within 30 days before study entry; laboratory abnormalities (alanine aminotransferase/aspartate aminotransferase/gamma-glutamyl transferase >2 × upper limit of normal [ULN]; neutrophils <1500/mm^3; lymphocytes <1000/mm^3; haemoglobin <10 g/dL; platelets <100/mm^3; prothrombin time >1.25 × ULN; creatinine clearance <70 mL/min according to the Cockcroft-Gault equation).

PDN Study

<u>*Inclusion criteria*</u>

Signed consent before study entry; willingness to understand and comply with protocol requirements; ≥18 years old; had pain due to polyneuropathy caused by type 1 or type 2 diabetes mellitus present for ≥6 months but <5 years, that began in the feet with relatively symmetrical onset; PDN diagnosis confirmed by a score of ≥3 on part B of the Michigan Neuropathy Screening Instrument (MNSI); moderate-to-severe pain intensity at screening and during the 7-day run-in period before Visit 2 (Day –1), defined as a mean score ≥4 on the NPRS; glycosylated haemoglobin (HbA1c) of ≤10.0% at screening; stable diabetes treatment within the last 3 months with no anticipated changes in medication regimen; male and female individuals were eligible – female patients of childbearing age were required to use an acceptable form of contraception during the study and up until 1 month after administration of the last dose of study medication; male patients were required to use an acceptable form of contraception if their female partner(s) was (were) pregnant or could become pregnant from the time of the first administration of the

study medication until 3 months following administration of the last dose of study medication.

Exclusion criteria

Pregnant or nursing; conditions that might have interfered with the assessment of the PDN (peripheral vascular disease, neurological disorders unrelated to diabetic neuropathy, other types of severe neuropathies, skin condition in the area of the neuropathy that could have altered sensation or other painful conditions); severe pain related to other causes; exposure to drugs known to cause neuropathy in the 30 days before receiving study medication; major psychiatric disorder; serious/unstable cardiovascular disease that could compromise participation or cause hospitalization; second/third degree atrioventricular blockade not corrected with a pacemaker or any clinically significant abnormality in the 12-lead electrocardiogram as determined by the investigator; taking specific drugs/drug classes that could not be discontinued – if discontinued, a minimum washout period of at least 5 × the half-life was required (benzodiazepines, skeletal muscle relaxants, orally administered steroids, centrally acting analgesics, opiates, topical lidocaine or capsaicin, non-steroidal anti-inflammatory drugs, anticonvulsants, tricyclic antidepressants, selective serotonin reuptake inhibitor or serotonin and norepinephrine reuptake inhibitors, N-methyl-D-aspartate antagonists, thalidomide, nitrous oxide, Ca2+/magnesium 2+ ion infusions); treated with a drug without regulatory approval for any indication in the 30 days before study entry; history of drug abuse/dependence within 1 year before the study; history of active serious medical conditions that could compromise safety/interfere with study assessments; history of drug abuse or dependence; history of severe gastroparesis/gastric bypass surgery, positive hepatitis B or C serology, or positive HIV test/known infection; previous neurolytic/neurosurgical treatment for neuropathic pain; malignancy in the past 2 years; active malignancy or injected anaesthetics/steroid use within 30 days before study entry; laboratory abnormalities (alanine aminotransferase/aspartate aminotransferase/gamma-glutamyl transferase >2 × ULN; neutrophils <1500/mm^3; lymphocytes <1000/mm^3; haemoglobin <10 g/dL; platelets <100/mm^3; prothrombin

time >1.25 × ULN; creatinine clearance <70 mL/min according to the Cockcroft-Gault equation).

S2. FULL LIST OF STUDY OUTCOMES AND DEFINITIONS FOR THE CPSP AND PDN STUDIES

Efficacy endpoints:

- Time-specific change from baseline to Day 28 in mean pain intensity in the previous 7-day interval measured by a NPRS included in a patient diary (average and worst 24-hour pain).
- 50% responder rates on Days 7, 14, 21 and 28, defined as the proportion of patients with a reduction from baseline of ≥50% in the mean 24-hour average pain score in the previous 7 days (measured by a NPRS included in a patient diary).
- 30% responder rates on Days 7, 14, 21 and 28, defined as the proportion of patients with a reduction from baseline of ≥30% in the mean 24-hour average pain score in the previous 7 days (measured by a NPRS included in a patient diary).
- Time-specific change from baseline to Days 7, 14 and 21 in mean pain intensity in the corresponding previous 7 days measured by a NPRS included in a patient diary (average and worst 24-hour pain).
- Time to onset of sustained therapeutic improvement, defined as first day on which patients demonstrated a ≥1-point reduction in mean NPRS score from baseline in patients with at least a 30% and 50% reduction in mean pain score on Day 28 (average and worst 24-hour pain).
- Percentage of patients needing rescue medication and amount of rescue medication used.
- Change from baseline to Day 28 in short-form McGill Pain Questionnaire.

- Change from baseline to Days 7, 14, 21 and 28 in short-form Brief Pain Inventory (SF-BPI).
- Change from baseline to Days 7, 14, 21 and 28 in Neuropathic Pain Symptom Inventory (NPSI).
- Change from baseline to Days 7, 14, 21 and 28 in allodynia and hyperalgesia, measured by visual analogue scale after stimulus (brushing and pinprick).
- Change from baseline to Days 7, 14, 21 and 28 in the pain, allodynia and hyperalgesia area (CPSP Study only).
- Assessment of post-treatment pain by measurement of efficacy endpoints (pain intensity measured by NPRS in patient diary, SF-BPI, NPSI, allodynia and hyperalgesia) at Day 35.
- Change from baseline to Day 28 in 36-item Short Form Health Survey (SF-36).
- Patient Global Impression of Change on Day 28.

Safety endpoints:

- Adverse events reported (spontaneous reporting by patients and from asking patients non-leading questions, e.g., “How do/did you feel?”).
- Percentage of patients reporting ≥1 adverse event.
- Laboratory tests at screening visit and Day 28.
- Vital signs and electrocardiogram findings at each visit
    - Systolic and diastolic blood pressure
    - Heart rate
    - Respiration rate
    - Body temperature.

S3. RANDOMIZATION

For both studies, patients were randomized to E-52862 or placebo in a 1:1 ratio using balanced randomization in blocks to ensure the same number of patients in each treatment group. The list of random numbers used to allocate the study treatment was generated using Statistical Analysis System© (CPSP Study: RPS Strategic Solutions; PDN Study: SAS Institute Inc.), which was independent of the study sponsor. The statistician responsible for the randomization list was not involved in the data management or statistical analyses of the study. The randomization list was sent directly to the manufacturer to prepare the medication kits, which were then labelled with a patient code identifier according to the list. Each patient randomized within an investigational site was assigned the medication kit with the lowest patient code available at the site. Thus, patients were identified through consecutive patient codes within their centre, in their order of inclusion in the study. These codes were used in randomization procedures and as a means of keeping the confidentiality of patient's identity.

SUPPLEMENTARY TABLES AND FIGURES

TABLE S1 CPSP Study investigators (SIGM-205).

**Principal investigator name**	**Site name**
Rafael Gálvez Mateos	Pain Unit, Hospital Universitario Virgen de las Nieves, Granada
Carme Busquets Julià	Pain Unit, Hospital Clínic i Provincial Barcelona, Barcelona
Josep Lluis Aguilar Sánchez	Pain Unit/Anesthesia, Hospital Son Llàtzer, Palma de Mallorca
Juan Carlos de la Pinta García	Anesthesiology Service, Fundación Jiménez Díaz – UTE, Madrid
Juan Carlos Tornero Tornero	Pain Unit/Anesthesiology Service, Hospital Clínico Universitario de Valencia, Valencia
Jerónimo Herrera Silva	Pain Unit, Hospital El Tomillar, Sevilla
Antonio Montes Pérez	Pain Unit/Anesthesiology Service, Hospital del Mar, Barcelona
José Luis Muñoz-Blanco	Neurology Service, Hospital General Universitario Gregorio Marañón, Madrid
Nelly Albesa Caro	Pain Unit, Policlínico Ruber, Madrid
Francisco Javier Medel Rebollo	Pain Unit, Hospital Vall d'Hebrón, Barcelona
Víctor Mayoral Rojals	Pain Unit, Hospital Bellvitge, Barcelona
César Margarit Ferri	Pain Unit, Hospital General Universitario de Alicante, Alicante
Luz Cánovas Martínez	Pain Unit, Complejo Hospitalario de Ourense, Ourense
Fernando Neira Reina	Anesthesia Unit, Hospital Universitario Puerto Real, Puerto Real
Dolores López Alarcón	Pain Unit/Anesthesiology Service, Hospital General de Valencia, Valencia

Abbreviations: CPSP, chronic postsurgical pain.

TABLE S2 PDN Study investigators (SIGM-204).

Principal investigator name	Site name
**Study centres in Spain**	
José María Gómez-Argüelles	Neurology Service, Hospital Virgen de la Luz, Cuenca
Antonio Guerrero Sola	Neurology Service, Hospital Clínico San Carlos, Madrid
Carmen Díaz	Endocrinology Service, Hospital General Universitario de Alicante, Alicante
Jerónimo Herrera	Pain Unit, Hospital El Tomillar, Sevilla
Sergio Zavala Plaza	Pain Unit/Anesthesiology Service, Hospital de la Ribera, Valencia
Enrique Ortega	Pain Unit, Hospital Rio Hortega, Valladolid
José Manuel Gónzalez Mesa	Pain Unit, Hospital Universitario Virgen de La Victoria, Málaga
Eduardo Esteve	Endocrinology Service, Hospital Universitari de Girona Dr. Josep Trueta, Girona
Carlos Martín-Estefania	Neurology Service, Hospital Clínico San Juan de Alicante, Alicante
**Study centres in Romania**	
Anca Cerghizan	Diabetes/Endocrinology Service, Spitalul Clinic Judetean Cluj-Napoca, Cluj-Napoca
Carmen Crisan	Medical Center Mediab SRL (Diabetes), Targu-Mures
Adriana Dumitrescu	Endocrinology Service, Centrul Medical 'Sanatatea Ta' SRL, Bucharest
Carmen Natea	Diabetes/Endocrinology Service, Spitalul Clinic Judetean de Urgenta Sibiu, Sibiu
Nicoleta Mindrescu	Diabetic Center, Nicodiab SRL, Bucharest
Alina Nicolau	Diabetic Center, Institutul National de Diabet, Nutritie si Boli Metabolice 'Prof. Dr. N.C. Paulescu', Bucharest

Adriana Onaca	Endocrinology Service, Spitalul Pelican Oradea, Oradea
Bogdan Popa	Endocrinology Service, Spitalul Clinic Judetean de Urgenta Ploiesti, Ploiesti
Alexandrina Popescu	Diabetes/Endocrinology Service, Diabmed Dr. Popescu Alexandrina SRL, Ploiesti
Daniela Ecaterina Popescu	Endocrinology Service, Kristef Med SRL, Craiova
Georgeta Vacaru	Diabetes/Endocrinology Service, EasyDiet SRL, Bucharest
Magdalena Morosanu	Diabetes Service, Cabinet Medical Individual Dr. Morosanu Magdalena, Galati

Abbreviations: PDN, painful diabetic neuropathy.

TABLE S3 Prior neuropathic pain medications for patients in the CPSP Study**.**

**Prior neuropathic pain treatment, *n* (%)**	**E-52862 *n* = 55**	**Placebo *n* = 59**
Amitriptyline	3 (5.5%)	6 (10.2%)
Amitriptyline hydrochloride, perphenazine	1 (1.8%)	0
Ascorbic acid, paracetamol	0	1 (1.7%)
Baclofen	1 (1.8%)	0
Betamethasone	1 (1.8%)	1 (1.7%)
Betamethasone acetate, betamethasone sodium phosphate	1 (1.8%)	0
Bromazepam	1 (1.8%)	0
Bupivacaine	2 (3.6%)	1 (1.7%)
Buprenorphine	0	1 (1.7%)
Capsaicin	10 (18.2%)	15 (25.4%)
Celecoxib	1 (1.8%)	0
Clavulanic acid, amoxicillin trihydrate	0	1 (1.7%)
Clonazepam	1 (1.8%)	4 (6.8%)
Clonidine	1 (1.8%)	0
Cyclobenzaprine	0	1 (1.7%)
Dexamethasone	1 (1.8%)	1 (1.7%)
Dexketoprofen	5 (9.1%)	2 (3.4%)
Diazepam	4 (7.3%)	2 (3.4%)
Diclofenac	5 (9.1%)	2 (3.4%)

Domperidone	0	1 (1.7%)
Duloxetine	12 (21.8%)	11 (18.6%)
Etoricoxib	1 (1.8%)	0
Fentanyl	2 (3.6%)	5 (8.5%)
Gabapentin	9 (16.4%)	12 (20.3%)
Hyoscine butylbromide, metamizole magnesium	1 (1.8%)	0
Ibuprofen	12 (21.8%)	9 (15.3%)
Ketorolac	1 (1.8%)	2 (3.4%)
Lacosamide	1 (1.8%)	0
Levobupivacaine	1 (1.8%)	0
Lidocaine	17 (30.9%)	20 (33.9%)
Lidocaine, methylprednisolone	0	1 (1.7%)
Lidocaine, prilocaine	3 (5.5%)	4 (6.8%)
Lorazepam	0	2 (3.4%)
Lormetazepam	1 (1.8%)	1 (1.7%)
Medical gases	1 (1.8%)	0
Meloxicam	0	1 (1.7%)
Mepivacaine	1 (1.8%)	0
Metamizole	18 (32.7%)	20 (33.9%)
Naloxone hydrochloride, oxycodone hydrochloride	1 (1.8%)	0

Naproxen	2 (3.6%)	3 (5.1%)
Omeprazole	1 (1.8%)	1 (1.7%)
Other local anaesthetics	2 (3.6%)	1 (1.7%)
Oxycodone	0	1 (1.7%)
Paracetamol	24 (43.6%)	30 (50.8%)
Paracetamol, codeine	0	1 (1.7%)
Paracetamol, tramadol hydrochloride	6 (10.9%)	4 (6.8%)
Pregabalin	27 (49.1%)	27 (45.8%)
Procaine	0	1 (1.7%)
Tapentadol	3 (5.5%)	5 (8.5%)
Tetrazepam	0	1 (1.7%)
Topiramate	1 (1.8%)	0
Tramadol	14 (25.5%)	15 (25.4%)
Tramadol, paracetamol	2 (3.6%)	3 (5.1%)
Trazodone	0	1 (1.7%)
Triamcinolone acetonide, lidocaine hydrochloride	0	1 (1.7%)

Abbreviations: CPSP, chronic postsurgical pain.

TABLE S4 Prior medications for patients in the PDN Study.

**Prior neuropathic pain treatment, *n* (%)**	**E-52862 *n* = 85**	**Placebo *n* = 78**
Allergen extracts	0	1 (1.3%)
Amides	1 (1.2%)	1 (1.3%)
Anilides	6 (7.1%)	3 (3.8%)
Antidepressants, non-selective monoamine reuptake inhibitors	1 (1.2%)	1 (1.3%)
Antidepressants, other	7 (8.2%)	1 (1.3%)
Benzodiazepine derivatives	2 (2.4%)	1 (1.3%)
Carboxamide derivatives	2 (2.4%)	1 (1.3%)
Combinations of vitamins	44 (51.8%)	41 (52.6%)
Coxibs	0	1 (1.3%)
Drugs used in opioid dependence	1 (1.2%)	0
Glucocorticoids	1 (1.2%)	0
Heparin group	1 (1.2%)	0
Natural opium alkaloids	3 (3.5%)	1 (1.3%)
Other opioids	4 (4.7%)	2 (2.6%)
Other analgesics and antipyretics	18 (21.2%)	21 (26.9%)
Plain vitamin preparations	2 (2.4%)	1 (1.3%)
Propionic acid derivatives	1 (1.2%)	1 (1.3%)
Pyrazolones	1 (1.2%)	0
Various alimentary tract and metabolism products	29 (34.1%)	27 (34.6%)

Vitamins, other combinations	0	1 (1.3%)
Vitamin B1	4 (4.7%)	3 (3.8%)
Vitamin B1 and B6	15 (17.6%)	11 (14.1%)
Vitamin B12	2 (2.4%)	1 (1.3%)

Abbreviations: PDN, painful diabetic neuropathy.

TABLE S5 Efficacy outcomes of subgroup analyses in the CPSP Study (per-protocol analysis set).

	**E-52862** ***n*** **= 41**	**Placebo** ***n*** **= 46**	**Difference in least squares mean (95% CI)**	***p*** **value**
Mean change in average NPRS score in the previous 7 days from baseline to Week 4 (SD)				
Age				
<65 years	–1.8 (2.2)	–0.7 (1.7)	–1.4 (–2.5, –0.3)	0.013
≥65 years	–0.9 (1.2)	–2.0 (2.2)	2.0 (–1.0, 5.0)	0.154
Pain localization				
Abdominal/inguinal	–2.2 (2.7)	–0.9 (2.1)	–1.5 (–4.2, 1.2)	0.262
Back	–2.1 (2.9)	–0.4 (1.5)	NE[a]	NE[a]
Head	–	–2.4 (0.5)	–	–
Lower limb	–1.0 (1.1)	–1.5 (2.1)	0.4 (–1.2, 2.1)	0.613
Lumbar	–1.1 (1.7)	–1.6 (2.0)	–0.1 (–3.2, 3.0)	0.925
Thorax	–2.3 (2.4)	–1.5 (1.6)	–0.5 (–4.2, 3.3)	0.780
Upper limb	–1.4 (1.7)	–1.1 (2.2)	–2.1 (–4.2, 0.1)	0.055
Mean change in worst NPRS score in the previous 7 days from baseline to Week 4 (SD)				

Age				
<65 years	–2.0 (2.5)	–0.9 (2.2)	–1.1 (–2.4, 0.1)	0.075
≥65 years	–1.7 (1.9)	–2.2 (2.3)	2.0 (–2.9, 6.9)	0.362
Pain localization				
Abdominal/inguinal	–2.1 (3.4)	–1.3 (2.1)	–0.6 (–3.7, 2.6)	0.706
Back	–2.4 (2.9)	–0.3 (1.9)	–	–
Head	–	–1.5 (2.1)	–	–
Lower limb	–1.3 (1.4)	–1.9 (3.0)	0.3 (–2.2, 2.8)	0.783
Lumbar	–1.1 (1.6)	–1.7 (3.2)	1.0 (–3.0, 5.1)	0.569
Thorax	–2.0 (2.4)	–1.5 (2.3)	1.5 (–2.8, 5.8)	0.412
Upper limb	–1.8 (1.9)	–0.7 (2.1)	–1.1 (–3.4, 1.1)	0.267

[a]Least squares means are not estimable for patients with back pain localization during the Day 22–28 period due to a non-positive residual variance estimate.

Abbreviations: CI, confidence interval; CPSP, chronic postsurgical pain; NE, not estimable; NPRS, numerical pain rating scale; SD, standard deviation.

TABLE S6 Patient global impression of change at Week 4 in the CPSP Study and the PDN Study (full analysis set).

	**CPSP Study**				**PDN Study**		
	**E-52862** ***n* = 55**	**Placebo** ***n* = 59**	***p* value**		**E-52862** ***n* = 85**	**Placebo** ***n* = 78**	***p* value**
PGIC in pain from study treatment start to Week 4, *n* (%)				PGIC in pain from study treatment start to Week 4, *n* (%)			
Very much better	7 (12.7)	2 (3.4)	0.311	Very much improved	8 (9.5)	11 (14.5)	0.716
Much better	13 (23.6)	11 (18.6)		Much improved	36 (42.9)	22 (28.9)	
A little better	12 (21.8)	19 (32.2)		Minimally improved	27 (32.1)	31 (40.8)	
No change	18 (32.7)	20 (33.9)		No change	9 (10.7)	8 (10.5)	
A little worse	2 (3.6)	3 (5.1)		Minimally worse	4 (4.8)	2 (2.6)	
Much worse	0	0		Much worse	0	1 (1.3)	
Very much worse	2 (3.6)	1 (1.7)		Very much worse	0	0	
Missing	1 (1.8)	3 (5.1)		Missing	0	1 (1.3)	

Abbreviations: CPSP, chronic postsurgical pain; PDN, painful diabetic neuropathy; PGIC, patient global impression of change.

TABLE S7 Change from baseline in allodynia and hyperalgesia VAS scores in the CPSP Study (full analysis set).

	**E-52862** ***n* = 55**	**Placebo** ***n* = 59**	**Difference in least squares mean (95% CI)**	***p* value**
Mean change in VAS allodynia score, mm, in the previous 7 days from baseline (SD)				
Baseline (mean [SD] absolute value)	56.9 (28.5)	63.5 (27.6)		
Day 7	0.9 (20.3)	–12.1 (29.8)		
Day 14	–5.3 (26.8)	–12.2 (35.1)		
Day 21	–9.1 (28.4)	–15.1 (31.6)		
Day 28	–14.0 (32.6)	–13.8 (33.2)	–3.9 (–15.4, 7.6)	0.500
Mean change in VAS hyperalgesia score, mm, in the previous 7 days from baseline (SD)				
Baseline (mean [SD] absolute value)	76.3 (14.7)	75.3 (23.4)		
Day 7	–3.3 (18.4)	–9.2 (26.6)		
Day 14	–12.0 (25.0)	–11.4 (30.3)		
Day 21	–16.2 (29.5)	–13.5 (32.1)		
Day 28	–19.7 (32.7)	–15.1 (33.6)	–4.5 (–16.2, 7.3)	0.455

Abbreviations: CI, confidence interval; CPSP, chronic postsurgical pain; SD, standard deviation; VAS, visual analogue scale.

TABLE S8 Efficacy outcomes of subgroup analyses in the PDN Study (full analysis set).

	**E-52862** *n* = 85	**Placebo** *n* = 78	**Difference in least squares mean (95% CI)**	***p* value**
Mean change in average NPRS score in the previous 7 days from baseline to Week 4 (SD)				
Age				
<65 years	–2.1 (1.8)	–2.1 (1.7)	–0.1 (–0.6, 0.5)	0.854
≥65 years	–2.5 (1.6)	–2.2 (2.2)	0.0 (–1.0, 1.0)	0.950
HbA1C levels				
>8%	–2.2 (1.7)	–2.3 (1.2)	0.4 (–0.7, 1.4)	0.475
≤8%	–2.1 (1.8)	–2.1 (2.0)	–0.2 (–0.7, 0.4)	0.596
Mean change in worst NPRS score in the previous 7 days from baseline to Week 4 (SD)				
Age				
<65 years	–2.3 (1.8)	–2.5 (1.8)	0.2 (–0.4, 0.9)	0.471
≥65 years	–2.4 (1.5)	–2.6 (2.2)	0.4 (–0.7, 1.4)	0.502

HbA1C levels				
>8%	–2.3 (1.9)	–2.7 (1.2)	0.4 (–0.8, 1.6)	0.529
≤8%	–2.3 (1.7)	–2.5 (2.1)	0.2 (–0.5, 0.8)	0.629

Abbreviations: CI, confidence interval; HbA1c, glycosylated haemoglobin; NPRS, numerical pain rating scale; PDN, painful diabetic neuropathy; SD, standard deviation.

TABLE S9 Change from baseline in allodynia and hyperalgesia VAS scores in the PDN Study (full analysis set).

	**E-52862** *n* = 85	**Placebo** *n* = 78	**Difference in least squares mean (95% CI)**	***p* value**
Mean change in VAS allodynia score, mm, in the previous 7 days from baseline (SD)				
Baseline (mean [SD] absolute value)	42.4 (25.6)	47.6 (25.0)		
Day 7	–4.1 (14.9)	–4.0 (19.0)		
Day 14	–8.3 (15.2)	–9.1 (18.2)		
Day 21	–10.2 (18.3)	–11.6 (22.6)		
Day 28	–15.1 (22.5)	–16.2 (24.3)	–1.1 (–6.1, 3.8)	0.653
Mean change in VAS hyperalgesia score, mm, in the previous 7 days from baseline (SD)				
Baseline (mean [SD] absolute value)	51.3 (24.9)	52.1 (23.1)		
Day 7	–10.4 (15.6)	–6.8 (16.3)		
Day 14	–13.9 (16.7)	–11.7 (17.5)		
Day 21	–18.0 (18.9)	–16.1 (22.2)		

Day 28	–19.9 (24.7)	–19.3 (24.8)	–0.5 (–5.7, 4.8)	0.863

Abbreviations: CI, confidence interval; PDN, painful diabetic neuropathy; SD, standard deviation; VAS, visual analogue scale.

FIGURE S1 Mean 24-hour average (a) and worst (a) pain intensity by NPRS over the previous 7 days in the CPSP Study in patients with spinal surgery pain (full analysis set).

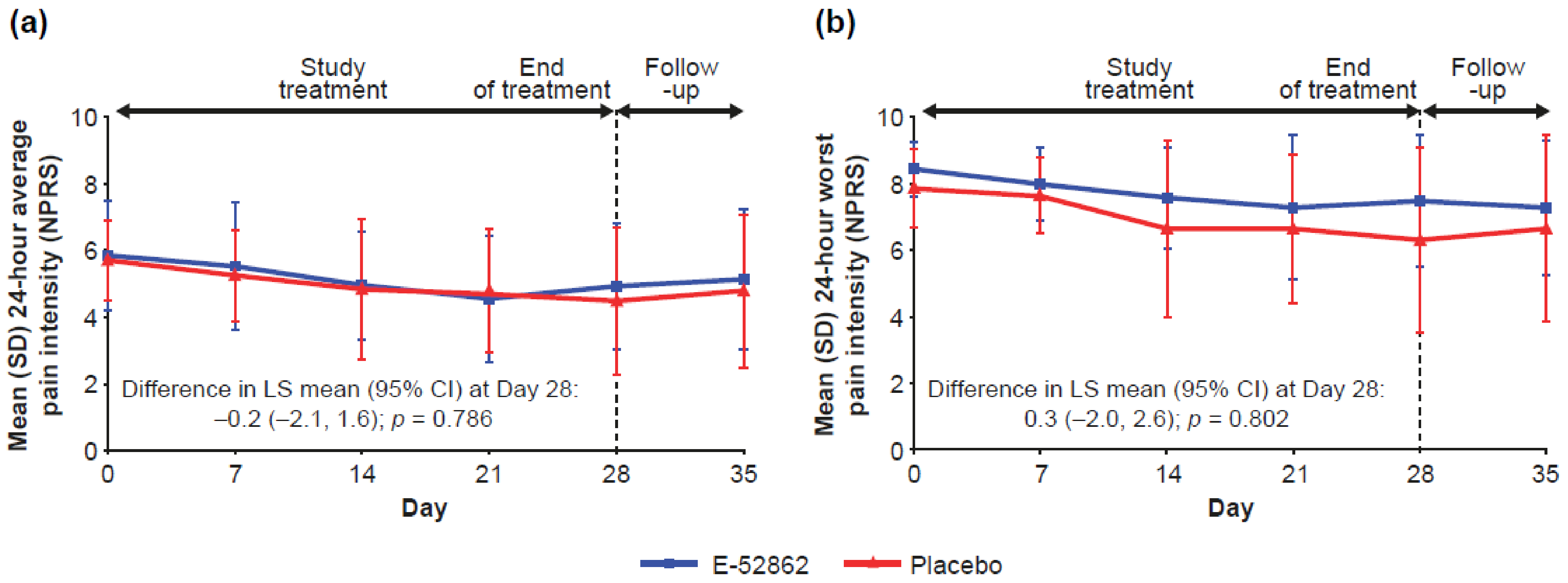

Abbreviations: CI, confidence interval; LS, least squares; NPRS, numerical pain rating scale; PDN, painful diabetic neuropathy; SD, standard deviation.

FIGURE S2 Mean NPSI domain and total intensity scores in the previous 7 days: superficial spontaneous pain (a), deep spontaneous pain (b), paroxysmal pain (c), evoked pain (d), paresthesia/dysesthesia (e), and NPSI total pain intensity score (f) in the CPSP Study (full analysis set).

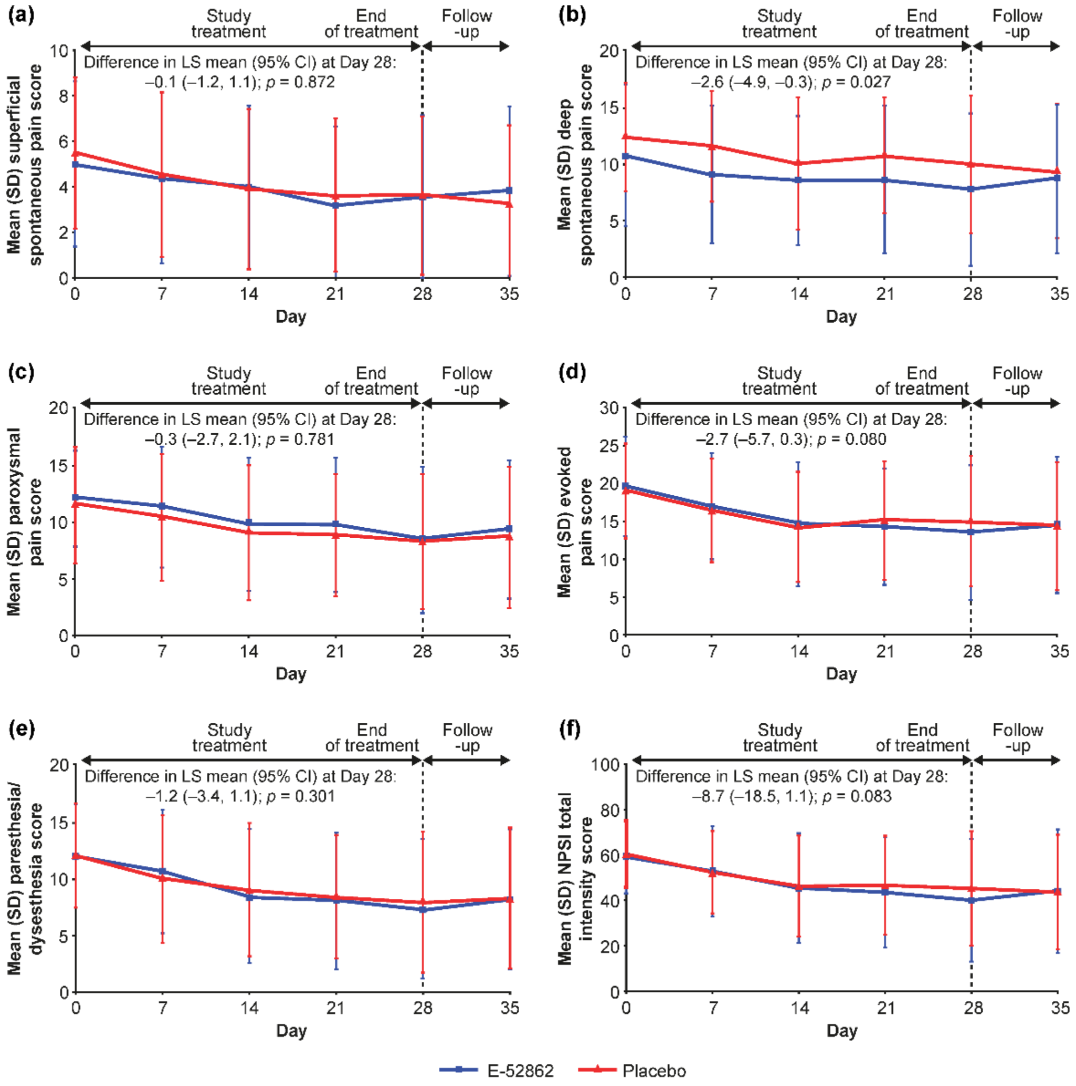

NPSI dimension score ranges: superficial spontaneous pain, 0–10; deep spontaneous pain, 0–20; paroxysmal pain, 0–20; evoked pain, 0–30; paresthesia/dysesthesia, 0–20. NPSI total pain intensity score ranges: 0–100.

Abbreviations: CI, confidence interval; CPSP, chronic postsurgical pain; LS, least squares; NPSI, Neuropathic Pain Symptom Inventory; SD, standard deviation.

FIGURE S3 Mean NPSI domain and total intensity scores in the previous 7 days: superficial spontaneous pain (a), deep spontaneous pain (b), paroxysmal pain (c), evoked pain (d), paresthesia/dysesthesia (e), and NPSI total pain intensity score (f) in the PDN Study (full analysis set).

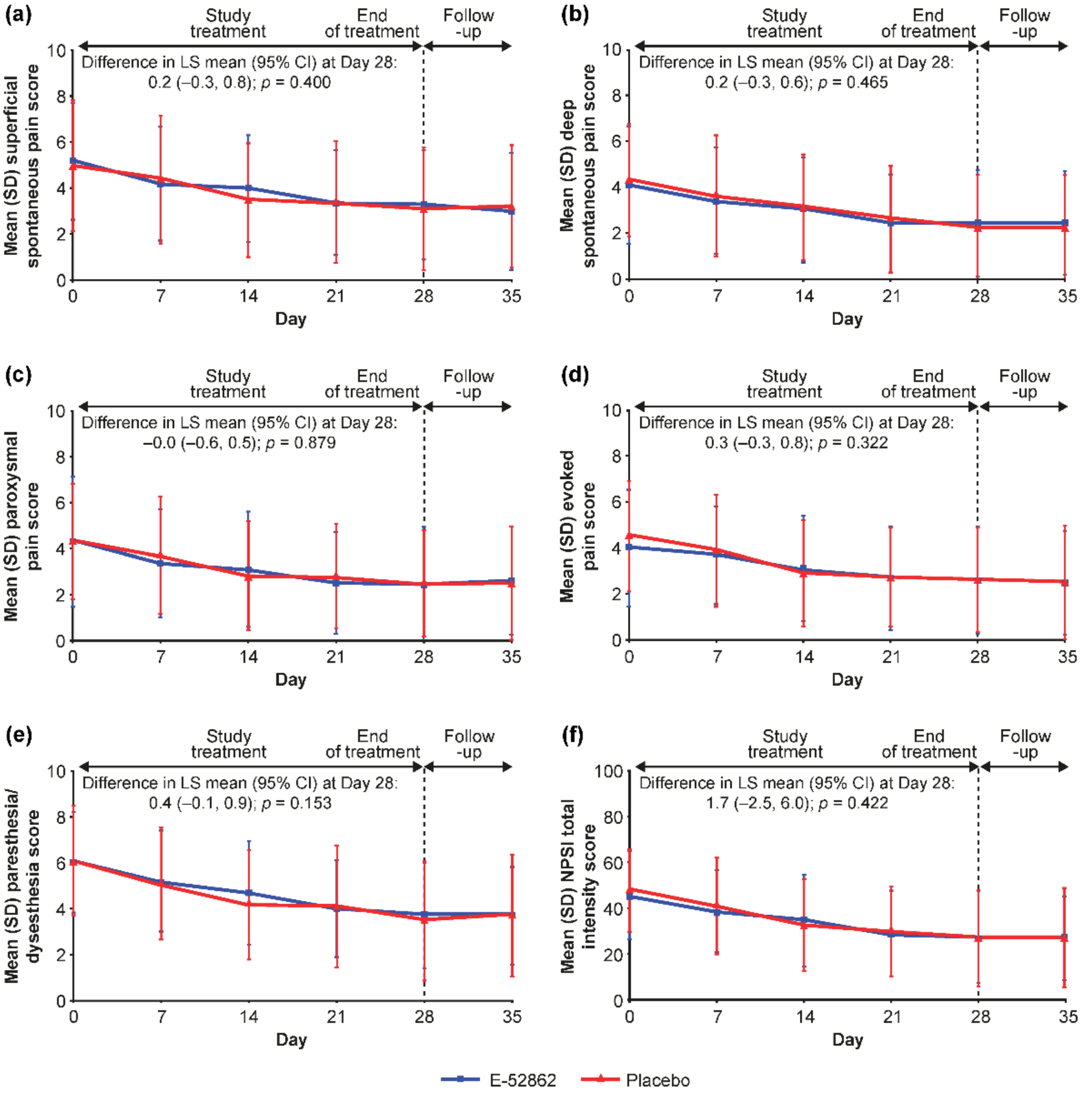

NPSI dimension score ranges: superficial spontaneous pain, 0–10; deep spontaneous pain, 0–10; paroxysmal pain, 0–10; evoked pain, 0–10; paresthesia/dysesthesia, 0–10. NPSI total pain intensity score ranges: 0–100.

Abbreviations: CI, confidence interval; LS, least squares; NPSI, Neuropathic Pain Symptom Inventory; PDN, painful diabetic neuropathy; SD, standard deviation.
